# Genome-wide CRISPR screen reveals PEX11B as a host restriction factor against ORFV through membrane fluidity regulation

**DOI:** 10.1371/journal.ppat.1013767

**Published:** 2026-07-15

**Authors:** Xiaoran Gao, Jinyang Hao, Sha Lu, Shida Wang, Yu Sun, Xin Ke, Xia Gao, Yuan Su, Yuze Sun, Yu Tian, Wenyu Yan, Jinliang Wang, Zhong Zheng, Rong Hai, Qianyi Zhang, Jingfei Wang, Wei Hu, Guojun Wang

**Affiliations:** 1 The State Key Laboratory of Reproductive Regulation and Breeding of Grassland Livestock, College of Life Sciences, Inner Mongolia University, Hohhot, P.R.China; 2 College of Basic Medical Sciences, Inner Mongolia Medical University, Hohhot, P.R. China; 3 The State Key Laboratory for Animal Disease Control, Harbin Veterinary Research Institute, Chinese Academy of Agricultural Sciences, Harbin, P.R. China; 4 Department of Microbiology and Plant Pathology, University of California, Riverside, California, United States of America; 5 China Institute of Veterinary Drug Control, Beijing, P.R. China; University of California, School of Medicine, UNITED STATES OF AMERICA

## Abstract

Host-pathogen interactions are shaped by cellular restriction factors that direct antiviral defenses. We built the first ovine genome-wide CRISPR knockout library in sheep testis (OA3.Ts) cells, targeting all protein-coding genes. Using this platform, we identified PEX11B, a peroxisomal membrane regulatory protein, as a strong restriction factor against orf virus (ORFV) infection. Removing PEX11B increased viral susceptibility and triggered severe cytopathic effects with membrane fusion and syncytia formation. Mechanistic studies showed that PEX11B knockout harmed peroxisomal integrity and disrupted lipid metabolism. This led to greater plasma membrane fluidity, creating a proviral environment that allowed more viral entry and replication. These results reveal a new antiviral function for PEX11B in blocking viral infection and underscore the importance of peroxisomal regulation in host-virus interactions.

## Author summary

Orf virus (ORFV) causes significant disease in sheep and other ruminants, posing threats to livestock health and agricultural productivity. Host cells deploy restriction factors to fend off viral infections, but the key players mediating defense against ORFV remain poorly understood.

Here, we developed an ovine genome-wide CRISPR knockout library to systematically identify genes regulating ORFV infection. This approach uncovered PEX11B—a protein governing peroxisome function—as a critical antiviral restriction factor. Loss of PEX11B renders sheep cells highly susceptible to ORFV, driving massive viral replication and cell fusion. Mechanistically, PEX11B deficiency damages peroxisomal integrity, disrupts lipid metabolism, and increases plasma membrane fluidity, creating a favorable environment for viral entry and spread. Our findings reveal a novel antiviral role for PEX11B and link peroxisome-mediated lipid regulation to host defense against ORFV. This work provides new insights into host-virus interactions and offers a valuable genetic tool for exploring antiviral mechanisms in sheep, with implications for developing targeted strategies to control ORFV infections.

## 1 Introduction

Orf virus (ORFV), responsible for contagious pustular dermatitis (also known as ecthyma contagiosum), was first discovered in 1920 and is now present in more than 70 countries across Africa, the Near and Middle East, and Asia, leading to significant economic losses each year [1–4]. Annually, ORFV infects about 2 million small ruminants worldwide, causing an overall death rate of 10–20%, which rises to over 90% in lambs under three months old. The virus often triggers outbreaks even among fully vaccinated herds [1, 5–7]. Since 2017, large-scale epidemic waves have been documented in Uruguay, Colombia, Morocco and Brazil, underscoring an expanding threat to animal husbandry and zoonotic public health [1, 8–11]. The substantial economic burden imposed by ORFV, which encompasses production losses, treatment costs, and trade restrictions, underscores the imperative need for a nuanced understanding of the molecular mechanisms governing its infection, pathogenesis, and interaction with the host immune system [3, 12–14].

The ORFV infection cycle initiates with viral attachment to as-yet-unidentified host cell receptors, followed by entry and cytoplasmic replication via the formation of distinctive intracytoplasmic viral factories [15, 16]. Following genome replication and virion assembly, progeny virions are released through lytic or non-lytic mechanisms. Intriguingly, ORFV exhibits broad tropism, infecting diverse cell types across multiple species, including sheep, goats, humans, and other mammals, suggesting the exploitation of conserved or multifactorial entry pathways [7, 11, 17–19]. Recent research has begun to uncover the viral components involved in ORFV entry and replication. However, the host cell factors and mechanisms that play roles in these processes remain largely unexplored [20, 21]. Unraveling these virus-host interactions could unveil novel targets for antiviral intervention, offering a strategic advantage over traditional approaches that focus solely on viral proteins, as host-directed therapies may reduce the likelihood of resistance emergence.

Genetic screening technologies have revolutionized the identification of host dependencies critical for viral replication, providing actionable insights for antiviral development [22, 23]. Early methodologies, such as RNA interference (RNAi) and insertional mutagenesis, enabled large-scale functional genomics screens [24, 25]. More recently, CRISPR/Cas9-based genome-wide knockout screens have emerged as a powerful tool for systematically interrogating host factors essential for infections caused by SARS-CoV-2, Ebola virus, West Nile virus, and other high-consequence pathogens [26–33]. Notably, analogous CRISPR screens in livestock species such as pigs challenged with Japanese encephalitis virus, cattle with Bovine Parainfluenza Virus Type 3, and poultry with Avian influenza virus have successfully identified host determinants [34–36]. While CRISPR screening technology has been applied in ovine research, such applications have largely been restricted to targeted screens (e.g., candidate gene screening covering 3 chromosomes) [37]. In contrast, genome-wide CRISPR-Cas9 loss-of-function screening—encompassing all chromosomes of the sheep genome—remains significantly underutilized in ovine cells.

Here, we address this gap by establishing OA3.Ts cells stably expressing Cas9 and performing the first ovine genome-wide CRISPR/Cas9 screen to identify host restriction factors against ORFV. Our screen pinpointed PEX11B, a peroxisomal membrane regulator, as a potent antiviral factor. Genetic ablation of PEX11B dramatically enhanced ORFV susceptibility, inducing cytopathic effects characterized by membrane fusion and syncytia formation. Mechanistically, PEX11B knockout disrupted peroxisomal integrity, dysregulating lipid metabolism and increasing plasma membrane fluidity—a proviral microenvironment facilitating viral entry and replication. These findings unveil PEX11B as a novel restriction factor and underscore peroxisomal regulation as a pivotal determinant of host-virus interactions, offering a platform for targeted interventions in livestock health.

## 2 Results

### 2.1 Development of an immortalized OA3.Ts-Cas9 cell line for genome-wide CRISPR screening

Despite the transformative potential of CRISPR screening in *Ovis* species for studying mammalian physiology and disease, progress has been hindered by the lack of expandable cell lines suitable for large-scale genetic interrogation. To address this bottleneck, we established an immortalized sheep testis (OA3.Ts) cell line stably expressing Cas9 and human telomerase reverse transcriptase (hTERT).

**Fig 1:**
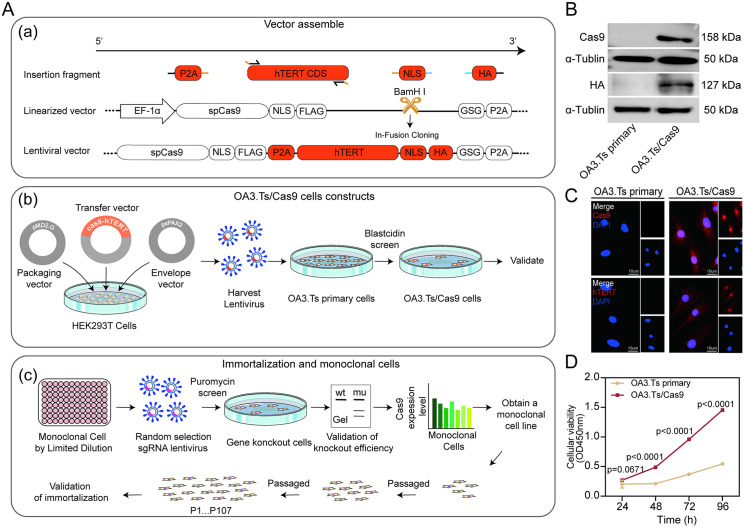
Establishment of immortalised OA3.Ts monoclonal cell lines stably expressing Cas9. (A) Comprehensive workflow for the generation and validation of immortalized OA3.Ts cell lines stably expressing Cas9: (a)Construction of the lentiviral expression vector Lenti-Cas9-hTERT, (b)Generation and validation of immortalized OA3.Ts cell lines stably expressing Cas9, (c)Verification and immortalization of monoclonal cells. (B) Western blot analysis of Cas9, HA, and α-Tubulin in OA3.Ts primary and OA3.Ts/Cas9 cells. α-Tubulin was used as a loading control. (C) Immunofluorescence staining of Cas9 and hTERT in OA3.Ts primary (Left panels) and OA3.Ts/Cas9 (Right panels) cells. Nuclei were counterstained with DAPI. Scale bar, 10 μm. (D) Proliferation curves of OA3.Ts primary and OA3.Ts/Cas9 cells determined by CCK-8 assay. Cell viability was quantified by measuring absorbance at OD450 nm using the CCK-8 kit at 24, 48, 72 and 96 h post seeding. Statistical differences between two cell groups at each time point were calculated, p values are indicated on the graph.

We engineered a lentiviral construct encoding hTERT—fused with P2A, NLS, and HA tags—and inserted it into a linearized lenti-blast-Cas9 plasmid via BamHI digestion (Figs 1A(a) and S1). Following lentiviral transduction, we generated OA3.Ts/Cas9 cells (Fig 1A(b)) and confirmed stable integration of Cas9 and hTERT by immunofluorescence and Western blotting (Fig 1B and 1C). Notably, the immortalized cells exhibited significantly enhanced proliferation compared to primary OA3.Ts cells (Fig 1D) and retained stable Cas9/hTERT expression over 107 passages, with minimal apoptosis (Figs 1A(c) and S2A-S2E), confirming their extended replicative capacity. To ensure functional compatibility with CRISPR screening, we isolated a monoclonal OA3.Ts/Cas9 line (clone #A) that maintained robust Cas9 expression and full susceptibility to ORFV infection (S3A-S3D and S4A–S4B Figs). To validate editing efficiency, we targeted the B4GALNT2 gene using lentiviral sgRNA delivery and observed stable CRISPR activity 6–10 days post-infection (S4C and S4D Fig). These results establish OA3.Ts/Cas9 as an ideal platform for genome-wide virus-host interaction studies.

### 2.2 Construction of an ovine genome-wide CRISPR/Cas9 knockout library

**Fig 2:**
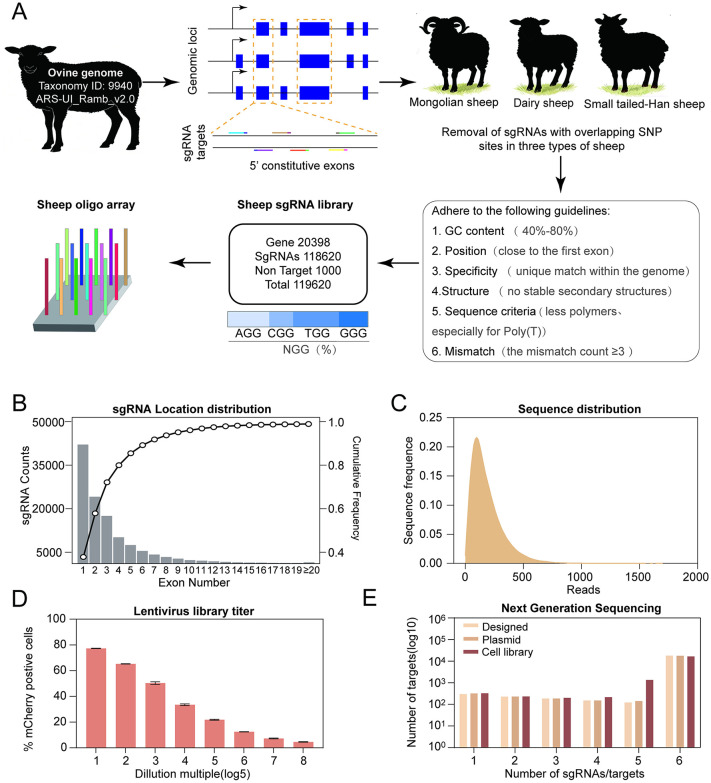
Generation of an ovine genome-wide CRISPR/Cas9 knockout library. (A) Schematic representation of the ovine genome-wide CRISPR/Cas9 knockout library design and construction workflow. Protein-coding genes from NCBI. (B) Distribution of cutting sites of all 118626 sgRNAs relative to the exon of across the genome. Bar chart showing the total number of gRNAs under the current exon. Line graph showing the proportion of frequencies under the current exon. (C) Sequence distribution of sgRNAs targeting sequences in plasmid-pooled sgRNA libraries.The frequency distribution of sgRNA counts from the transduction plasmid-pooled sgRNA libraries, illustrating the representation and uniformity of sgRNA coverage. (D) Genome-wide lentiviral library titer assay. Characterization by flow cytometry to detect the proportion of cells with red fluorescence. All sgRNAs were inserted into the Lenti-CRISPR-puro-sgRNA-mCherry expression vector. (E) The number of sgRNAs per gene in the genome-wide CRISPR pooled sgRNA library from the designed, plasmid, or mutant cell pools. The sgRNA was designed by the CRISPR-offinder software. Plasmid, the sequencing result of the sgRNA library from plasmid pools; Cell library, the sequencing result of the sgRNA library from sorted mutant cell populations.

We developed a comprehensive ovine genome-wide CRISPR/Cas9 knockout library targeting 20,398 protein-coding genes (Fig 2A). The sgRNAs (single guide RNAs) were designed against all RefSeq transcript isoforms in the Ovis aries reference genome (ARS-UI_Ramb_v2.0), prioritizing 20-bp sequences with 3′-NGG PAM motifs in exonic regions. To enhance breed compatibility, we excluded sgRNAs showing sequence divergence across three major sheep breeds (Mongolian, Dairy, and Small-tailed Han sheep). Using CRISPR-offinder (v1.2), we designed 118,620 high-specificity sgRNAs, supplemented with 1,000 non-targeting negative controls (S5A Fig and S1 Table). Guide RNAs were optimized for upstream exon targeting (Fig 2B) and minimal off-target activity (S5B Fig).

Deep sequencing of PCR-amplified sgRNA constructs confirmed 99.97% (119,566/119,596) guide representation (Figs 2C and S5C). Validation assays on three host genes confirmed efficient indel induction (up to 65.27% cutting efficiency) at intended loci (S5D Fig).The library demonstrated exceptional uniformity (Gini Index = 0.08234), far below the 0.2 threshold for balanced representation. Although minor abundance variations were observed, 99.76% of sgRNAs fell within a 10-fold frequency range, underscoring robust library quality.

For functional screening, we transduced OA3.Ts/Cas9 cells with the lentiviral library at a low MOI(multiplicity of infection) (≈0.2), achieving 20–30% transduction efficiency to ensure single-guide integration (Figs 2D and S5E). The resulting cell library retained 98.61% (117,958/119,620) of sgRNAs with high uniformity (Gini Index = 0.1151; Fig 2E), establishing ovine-GeCKO as a robust resource for ovine functional genomics.

### 2.3 Genome-wide CRISPR screen identifies host factors restricting ORFV infection

**Fig 3:**
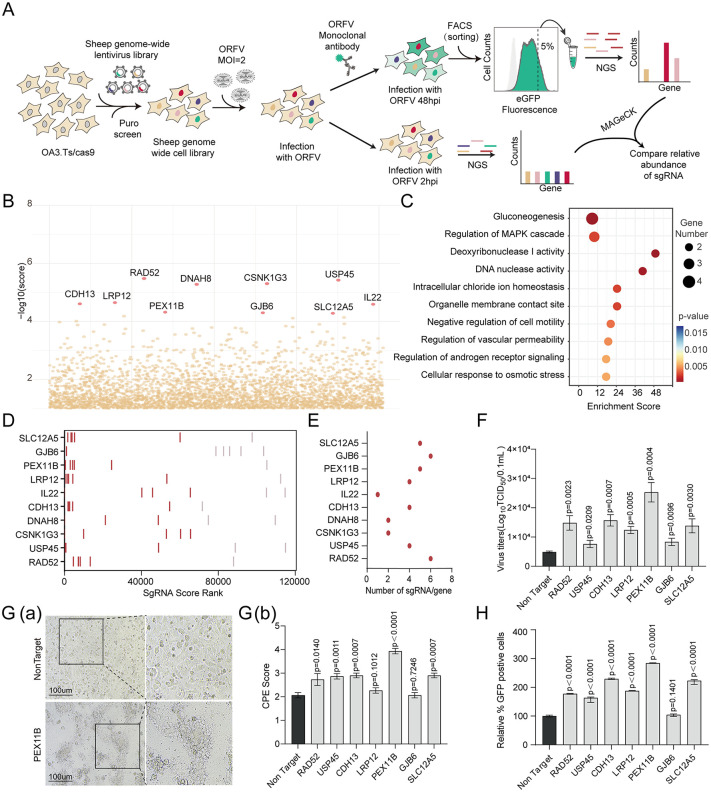
Identification of candidate genes resistant to ORFV infection via genome-wide CRISPR screen. (A) Schematic overview of FACS-based screening strategy. The sheep genome-wide CRISPR knockout library of OA3.Ts/Cas9 cells was infected with ORFV of MOI = 2. After 48hpi, a FACS-based positive selection for cells was performed. Deep sequencing identified candidate genes. (B) Spatial distribution of CRISPR knockout signals on the genome for the FACS-based positive screens. The core set of the Top ten robust hits is shown in red. (C) Gene Ontology (GO) Enrichment Analysis of the Top 0.1% candidate genes. (D) Top ten candidates ranked by MAGeCK enrichment score (higher rank indicates greater enrichment). The six guides for each candidate are ranked by individual enrichment. Enriched sgRNAs with Benjamini-Hochberg corrected Wald test *p* < 0.001 are colored in deepred.The light red sgRNAs represent values with *p* > 0.001. (E) The sgRNAs of the top ten candidates are summarised by the number of good sgRNAs in MAGeCK. (F) Seven knockout polyclonal cells were infected at MOI = 0.01 ORFV. The supernatant virus titre was determined 48 hours after infection. (G) (a) Cytopathic effect (CPE) images of Non target versus KO-PEX11B cells following infection with ORFV (MOI = 2) at 36 hours post-infection. Scale bar = 100 µm. (b)The viral Cytopathic effect (CPE) scoring plot of seven knockout polyclonal cells infected with ORFV at an MOI of 2 is presented. The scoring is based on the degree of lesions: 0: no lesions. 1: less than 25% cellular lesions. 2: 25–50% cellular lesions. 3: 50–75% cellular lesions. 4: more than 75% cellular lesions. (H) Flow cytometry-based relative quantitative analysis of ORF086 fluorescence intensity in seven ORFV-infected polyclonal cell lines.

To systematically identify host genes conferring resistance to ORFV infection, we performed a FACS-based (fluorescence-activated cell sorting) genome-wide CRISPR/Cas9 knockout screen. Cells from the knockout library were infected with ORFV (MOI = 2), and the top 5% of cells exhibiting high viral protein expression were sorted(Fig 3A). Genomic DNA from sorted and control cells underwent sgRNA amplification and next-generation sequencing. MAGeCK analysis revealed sgRNAs significantly enriched in infected cells, pinpointing antiviral host factors. The top ten most enriched candidate genes after ORFV infection were (from highest to lowest) RAD52, USP45, CSNK1G3, DNAH8, CDH13, IL22, LRP12, PEX11B, GJB6, and SLC12A5 (Fig 3B and S2 Table). We observed enrichment for 0.1% Top genes involved in regulation of MAPK cascade, which were also represented in previous genome-wide screens for other viruses [38] (Fig 3C and S3 Table). To narrow the focus for further analysis, we created a score ranking of all sgRNAs for the top ten most enriched genes from the screening strategy, as well as the number of good sgRNAs determined to be statistically significant based on statistical analysis (Fig 3D and 3E), we generated polyclonal knockout lines for functional validation (S6A Fig).

We next assessed ORFV infection dynamics in polyclonal knockout lines for the seven candidate genes. Knockout of all seven top candidate genes augmented ORFV infection, with PEX11B depletion showing the strongest effect (Fig 3F). In particular, ORFV infection 36 hour post infection associated with the KO-PEX11B(PEX11B knockout cells) showed a significant number of membrane fusion events within the cytopathic effect (CPE) (Figs 3G and S6B), corroborated by elevated infected cells and viral protein expression via immunofluorescence (S6C Fig) and flow cytometry (Figs 3H and S6D), implicating these genes as critical ORFV barriers.

### 2.4 PEX11B is a broad-spectrum gatekeeper of viral infection

Among the seven highest-ranked genes identified in the genome-scale CRISPR screen for candidates associated with ORFV infection, PEX11B, a gene implicated in peroxisome proliferation [39–41], was highlighted. In monoclonal PEX11B knockout cells, Western blott analysis and RT-qPCR confirmed the complete absence of both target protein and its corresponding mRNA expression(S7A-S7C Fig). Sanger sequencing and T7EI analysis confirmed the absence of off-target editing in the monoclonal cell line(S7D and S7E Fig).

**Fig 4:**
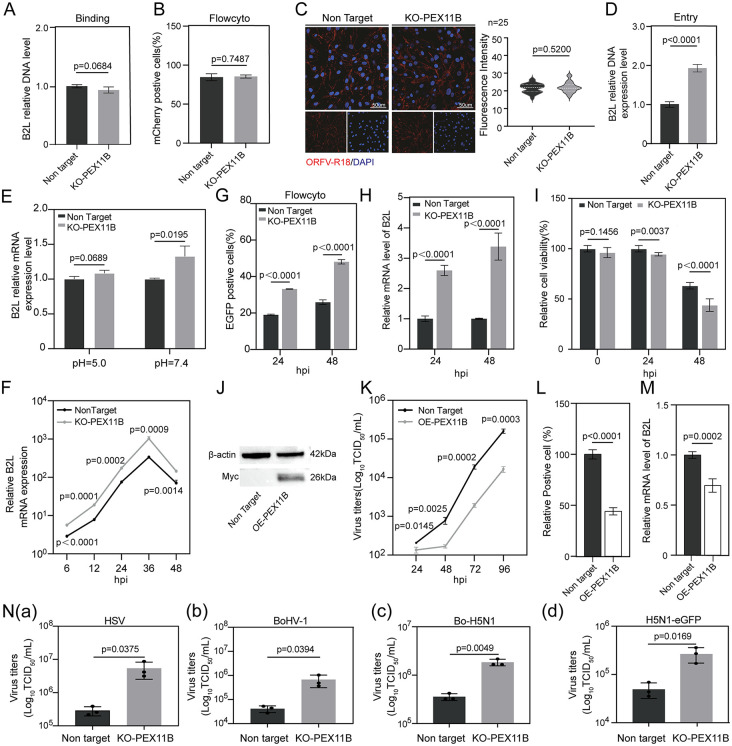
Identification of viral life-cycle defects in clonal PEX11B knockout cells. (A) The relative DNA abundance of the ORFV B2L gene as a readout of viral binding was compared between Non target cells and KO-PEX11B cells. (B) The percentage of mCherry-positive cells, representing binding ORFV-R18 (red), was analyzed by flow cytometry in Non target and KO-PEX11B cells. (C) Representative immunofluorescence images showing ORFV-R18 (red) bound to Non target and KO-PEX11B cells. Nuclei were counterstained with DAPI (blue). Scale bar = 50 μm. Violin plot showing the quantification of ORFV-R18 fluorescence intensity per cell (n = 25 cells per group). (D) Quantitative analysis of ORFV internalization. Non target and KO-PEX11B cell lines were infected with ORFV (MOI = 1) for 1.5 h at 37°C, and virus internalization was analyzed by qPCR. (E) Relative expression of the ORFV B2L gene was measured in Non target and KO-PEX11B cells at pH 5.0 and pH 7.4. Data are shown as mean ± SD. (F) Quantitative analysis of B2L gene expression levels at different time points after ORFV infection. (G) Flow cytometric analysis of infected-positive cell proportions in Non target and KO-PEX11B cells at 24 and 48 hours post-infection (hpi). Unpaired t-test, *p* < 0.0001 for both time points. (H) Relative ORFV mRNA levels in Non target and KO-PEX11B cells at 24 and 48 hpi (normalized to an endogenous control). Unpaired t-test, *p* < 0.0001 for both time points. (I) Relative cell viability of Non target and KO-PEX11B cells at 0, 24, and 48 hpi after ORFV infection at MOI = 5. (J) Western blot analysis of Myc-flag expression in Non target cells and PEX11B over-expression cells. (K) Non target and PEX11B over-expression lines were challenged with ORFV (MOI = 0.001), and the viral titers were measured at the indicated time post-infection. (L,M) Non target and PEX11B over-expression cell lines were challenged with ORFV at 24 hours post-infection. Fluorescence of the ORFV on the cell was shown in (L), and relative expression of ORFV was measured in (M). (N) Virus titer assays for Non target and KO-PEX11B cells infected with some virus. (a)HSV (MOI = 0.1, 24 hpi). (b) BoHV-1 (MOI = 1, 24 hpi). (c) Bo-H5N1 (MOI = 0.25, 24 hpi).(d) VN/H5N1 (MOI = 0.25, 24 hpi).

To pinpoint the infection stage modulated by PEX11B, we systematically interrogated the ORFV life cycle. Viral attachment remained unaltered in KO-PEX11B cells, as demonstrated by equivalent B2L envelope protein and ORF035 early gene binding (Figs 4A and S7F) and consistent flow cytometry/IFA results (Fig 4B and 4C). Strikingly, PEX11B ablation increased viral genome internalization by 1.5 hpi (Figs 4D and S7G), while acid-bypass assays confirmed its specific role in endocytosis-dependent entry (Fig 4E). B2L mRNA levels surged in KO-PEX11B cells by 6 hpi (Fig 4F), collectively establishing PEX11B as a critical checkpoint for early ORFV invasion. Post-entry, KO-PEX11B group increased infected cells (24–48 hpi; MOI = 5) (Fig 4G) and viral mRNA (Fig 4H), exacerbating cell death (Fig 4I). In contrast, PEX11B over-expression (OE) suppressed ORFV titers, infection rates, and B2L expression (Fig 4J–4M), confirming antiviral activity.

To determine whether PEX11B’s antiviral function extends beyond ORFV, we challenged KO cells with phylogenetically distinct viruses: the DNA viruses HSV-1 and BoHV-1(Bovine herpesvirus 1), and RNA viruses BoH5N1(Bovine H5N1) and VN/H5N1 (Influenza A) (Fig 4N). All viruses exhibited significantly enhanced replication in KO cells, demonstrating PEX11B’s capacity to restrict diverse viral families.

Our systematic analyses position PEX11B as a central hub of an underappreciated host defense pathway that limits infection by multiple viral families. The conservation of this restriction across DNA and RNA viruses suggests PEX11B may represent an evolutionarily ancient antiviral mechanism co-opted by diverse viral pathogens.

### 2.5 Peroxisome biogenesis is a proviral factor for ORFV replication

**Fig 5:**
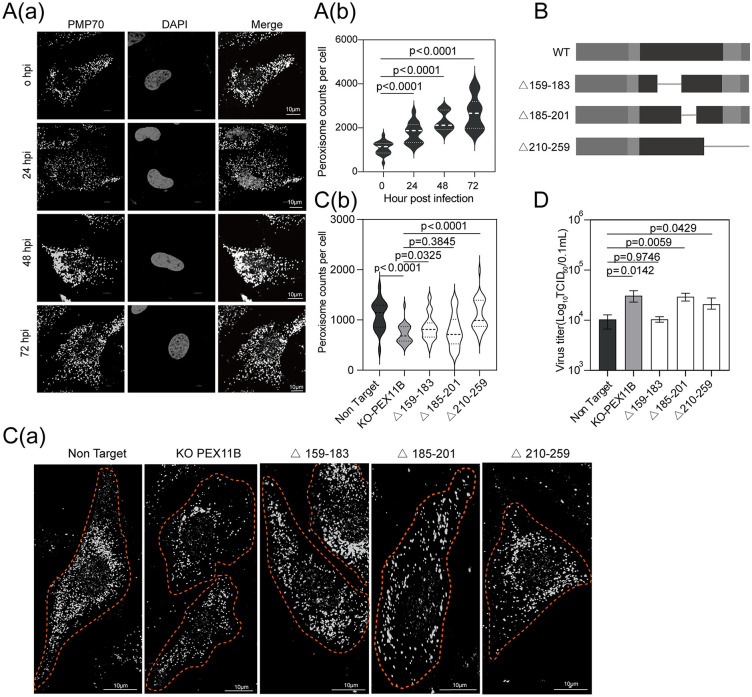
Antiviral function of PEX11B mediated by the action of peroxisomes. (A) Peroxisome numbers in ORFV-infected OA3.Ts/Cas9 cells. (a) Representative immunofluorescent images of OA3.Ts/Cas9 cells at 0, 24, 48, and 72 h post-infection (hpi) with ORFV (MOI = 0.5). Peroxisomes were labeled with anti-PMP70, and nuclei were counterstained with DAPI. Scale bar = 10 μm. (b) Quantification of peroxisome counts per cell at the indicated time points. N ≥ 20 cells. (B) Schematic of wild-type (WT) PEX11B and the PEX11B mutants △210–259,△159–183,△185–201.Stable complemented cell lines were established by lentiviral transduction of these truncated PEX11B fragments into KO-PEX11B cells. (C) (a)Representative images of peroxisome morphology (anti-PMP70) in Non target, KO-PEX11B cells, and PEX11B mutantsΔ159–183,Δ185–201 cells,Δ210–259. (b)Statistical summary of the number of peroxisomes quantified. Non Target N = 26793, PEX11B KO cells N = 19407, PEX11B mutants(Δ159–183) N = 27353,PEX11B mutants(Δ185–201) N = 24201,PEX11B mutants(Δ210–259) N = 33297. Scale bar = 10 µm. (D)ORFV titers in PEX11B truncation mutants. Viral titers were determined following ORFV infection (MOI = 0.01, 48 h post-infection) in Non target cells, KO-PEX11B cells, and three PEX11B truncation mutants (Δ159–183, Δ185–201, Δ210–259).

PEX11B orchestrates multiple stages of peroxisomal growth and division, including pre-fission membrane remodeling/elongation and division machinery assembly [42]. To delineate the role of peroxisomes in ORFV infection, we first analyzed the spatiotemporal relationship between viral replication and peroxisome dynamics via immunofluorescence microscopy. ORFV infection significantly increased peroxisome abundance (Fig 5A), indicating a potential functional link between peroxisome proliferation and viral replication.

To dissect its antiviral role, we generated PEX11B truncation mutants (Δ159–183, Δ185–201, and Δ210–259) via lentiviral transduction of truncated fragments into PEX11B knockout cells for stable complementation (Figs 5B and S7H). Intriguingly, PEX11B deletion markedly reduced peroxisome abundance per cell, and rescue with the Δ185–201 truncation failed to restore this phenotype, whereas Δ159–183 and Δ 210–259 partly reversed peroxisome loss (Fig 5C). Loss of PEX11B elevated ORFV titers; Δ159–183 could not rescue this proviral phenotype, while Δ185–201 and Δ 210–259 significantly promoted viral yields(Fig 5D). To further dissect the role of peroxisome biogenesis mediated by PEX11B in ORFV replication, we performed functional assays using PEX3 and PEX5 knockout cells (S10A-S10C Fig). Viral replication was markedly suppressed in both cell lines (S10D Fig), confirming that peroxisome biogenesis is required for efficient ORFV propagation. Together, these results reveal that PEX11B utilizes distinct structural modules to regulate peroxisome dynamics and to carry out its antiviral function.

### 2.6 PEX11B regulates peroxisomal morphology

**Fig 6:**
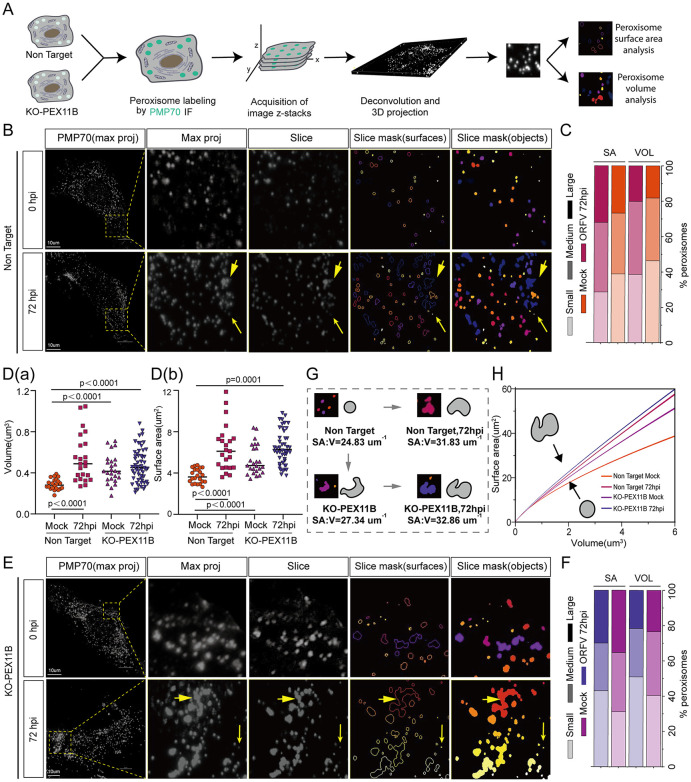
PEX11B knockout alters peroxisomal Membrane-to-Lumen Ratio to enhance ORFV infection. (A) Workflow for the analysis of peroxisome surface area and volume during viral infection. (B) Mock (top) and ORFV-infected (72 hpi, lower) Non Target cells labeled with anti-PMP70. ROI squares (left) show z stack maximum projection, two slice mask (surfaces and objects), and masks of peroxisomes from object analysis. Small peroxisomes (arrows) and enlarged peroxisomes (arrowheads) are indicated. Scale bar = 10 µm. (C) Analysis of peroxisome size distribution in mock and ORFV-infected (72 hpi) cells. Peroxisome surface area (SA) and volume (VOL) were quantified and binned into small (SA, < 0.9 μm^2^; VOL, < 0.06 μm^3^), medium (SA, 1.1-3.3 μm^2^; VOL, 0.07-0.34 μm^3^), and large (SA, > 3.3 μm^2^; VOL, > 0.34 μm^3^) categories. N = 8809 peroxisomes in mock; N = 10741 peroxisomes in ORFV. (D) The average volume of peroxisomes per cell is shown in (a), the average surface area of peroxisomes per cell is shown in (b). N = 22 cells in Non Target Mock; N = 21 in Non Target 72 hpi; N = 27 in KO-PEX11B Mock; N = 34 in KO-PEX11B 72 hpi. (E) KO-PEX11B cells in mock and infected conditions, as in (B).Scale bar = 10 µm. (F) Analysis of peroxisome size distribution in mock and ORFV-infected KO-PEX11B cells, as in (C). N = 9446 in KO-PEX11B Mock; N = 22641 in KO-PEX11B 72 hpi. (G) Peroxisome morphology changes during ORFV infection. Examples of peroxisome masks from 3D analysis are shown, with SA and VOL values indicated. (H) Surface area-to-volume ratio (SA/V) regression curves from data in (D), plotted in the physiologically relevant range of observed mock peroxisome volumes (1–6 μm^3^). Significance of *p* < 0.001 was determined by chi-squared test for (C) and (F) Significance was determined by Student’s t test for (D), marked with p. Error bars indicate SEM.

Following ORFV infection, the quantity of peroxisomes in cells rises, suggesting that this increase aids in viral replication. Beyond changes in number, the morphology of peroxisomes also plays an essential role in mediating their functions [43]. To explore this matter further, we utilized IF microscopy and 3D reconstruction technology to investigate peroxisome morphology during infection(Fig 6A). In uninfected Non target cells, peroxisomes exhibited uniform spherical shapes (Fig 6B). For Non target cells, at 72 hpi, we observed both enlarged and slightly smaller peroxisomes, analysis of individual peroxisomes revealed that the size distribution, measured by surface area and volume, becomes polarised late in infection and significantly increased at 72 hpi (Fig 6C and 6D). Surprisingly, peroxisomes in KO-PEX11B cells exhibited deformation and enlargement(Figs 6E and S9A). Likewise, after 72 hours of infection in KO-PEX11B cells, there was a significant increase in the number of peroxisomes, which not only grew in size but also displayed an unusual flattened shape(Fig 6E and 6F). As with other organelles, peroxisomes modify shape to increase membrane surface area for functional processes [44]. To assess whether morphological changes facilitate membrane expansion, we calculated the surface area-to-volume ratio (SA/V) of individual peroxisomes. Regular spheres have lower SA/V than irregular structures (e.g., tubules) of similar volume, so we hypothesized that irregular peroxisomes would exhibit higher SA/V ratios. ORFV infection significantly elevated the average peroxisome SA/V ratio (Fig 6G), and quadratic curve fitting revealed a positive correlation between SA/V at 72 hpi (Figs 6H and S9B). Meanwhile, To further investigate the relationship between peroxisomes and viral infection, we employed GW6471 and Wy14643 to regulate peroxisomal proliferation. [45, 46]. The results indicate that GW6471 treatment reduced ORFV titers, whereas Wy14643 significantly elevated viral yields (S10A-S10F Fig). Consistently, GW6471-treated cells displayed fewer peroxisomes alongside a lowered peroxisomal SA/V ratio, while Wy14643 exposure increased both peroxisome abundance and SA/V ratio(S10G-S10J Fig). Taken together, these results reveal that PEX11B deletion leads to the formation of enlarged, flattened peroxisomes with an elevated membrane-to-lumen ratio, ultimately promoting viral infection.

### 2.7 PEX11B deficiency reprograms lipid metabolism to elevate membrane fluidity

**Fig 7:**
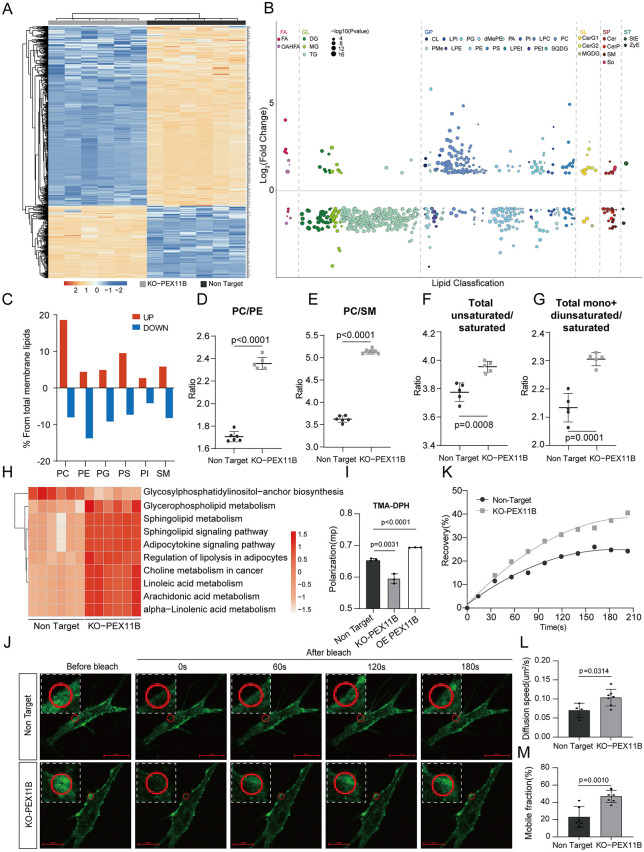
Knockout of PEX11B promotes cell membrane fluidity. (A) Heatmap showing the lipidomic analysis of Non target and KO-PEX11B cells. Each rectangle represents an ion feature colored by its normalized intensity scale from red (decreased level) to yellow (increased level). The dendrogram at the top was constructed based on the lipid intensity (similarity measure using Euclidean distance and the Ward clustering algorithm). (B) Global lipidomic profiling displays altered lipid abundance across major lipid classes upon PEX11B knockout.This dot plot visualizes the log2 fold change of individual lipid species (y-axis) grouped by major lipid classifications (x-axis). Dot size corresponds to −log10(p value), representing statistical significance of differential abundance. Distinct color coding differentiates subclasses within FA, GL, GP, SL, SP, and ST lipid families. Lipids positioned above the y = 0 line are significantly upregulated, while those below y = 0 are downregulated in KO-PEX11B cells relative to control cells. (C) Changes in cell-associated membrane lipid composition in KO-PEX11B cells relative to Non target cells. Red = percentage of upregulated lipids, blue = percentage of downregulated lipids per lipid subclass (PC, PE, PG, PS, PI, SM). Y-axis shows the percentage of altered lipids relative to total membrane lipids. (D,E) Quantification of PC/PE and PC/SM ratios in Non target control and KO-PEX11B cells. Scatter plot showing the molar ratio of PC to PE (D) and PC to SM (E) in Non Target and KO-PEX11B groups. Both ratios were markedly elevated upon PEX11B deletion. Statistical comparisons versus the Non target control yielded p < 0.0001 for each measurement. (F,G) Unsaturated-to-saturated fatty acid ratios in Non target and KO-PEX11B cells. (F) total unsaturated versus total saturated lipids; (G) total mono+diunsaturated versus total saturated lipids. (H) KEGG pathway analysis of bulk LC-MS data with averaged lipid expression shows the dynamic changes of lipid metabolism pathways across Non target cells and KO-PEX11B cells. (I) Fluorescence polarization (P) value changes of TMA-DPH-stained Non target cells, KO-PEX11B cells, and PEX11B over-expression cells. P was measured at an excitation wavelength of 355 nm and an emission wavelength of 430 nm. Data were collected from three parallel experiments. P is depicted as means ± SD, n = 3. (J) Fluorescence recovery images of Non target cells (Top) and KO-PEX11B cells (Bottom) after photobleaching upon various treatments. The radius of the selected region is 2.5 μm for all images. Scale bar = 20 μm. (K) Plots of fluorescence intensity of the Non target cells and KO-PEX11B cells of the marked area in (J) versus time after photobleaching. (L) Diffusion speed (μm^2^/s) of DIOC18-labeled cell membrane obtained from plots of normalized FRAP data. The means and SDs were from at least three different samples. (M) Mobile fraction (%) of DIOC18-labeled cell membrane obtained from plots of normalized FRAP data. The means and SDs were from at least three different samples.

Given peroxisomes’ central role in lipid metabolism [43, 47], we performed LC-MS lipidomics on KO-PEX11B cells and identified 624 dysregulated lipids (445 upregulated, 179 downregulated; *p* < 0.01). There were pronounced accumulations of triglycerides (TG) and phosphatidylcholine (PC). In contrast, we also observed depletions of phosphatidylethanolamine (PE) and sphingomyelin (SM) (Fig 7A–7C and S11A-S11C). Notably, PEX11B ablation led to increased ratios of PC:PE and PC/sphingomyelin (SM) (Figs 7D–7E and S11D-S11E), a perturbation known to reduce lipid packing density and increase membrane permeability [43, 47, 48]. Furthermore, KO-PEX11B cells exhibited a higher total unsaturated-to-saturated and mono- and diunsaturated-to-saturated fatty acid ratio (Fig 7F and 7G), suggesting enhanced desaturation that promotes membrane fluidity by increasing acyl chain disorder. Finally, heatmap analysis implicated PEX11B loss in activating the glycerophospholipid/sphingolipid metabolic pathway (Fig 7H), thus directly linking peroxisomal dysfunction to altered membrane properties.

To test the hypothesis that PEX11B knockout increases membrane fluidity through altered phospholipid balance and fatty acid unsaturation, we performed biophysical assays. TMA-DPH fluorescence polarization, which measures membrane rigidity by tracking fluorophore rotation, revealed significantly reduced anisotropy (P = 0.595 ± 0.002) in KO-PEX11B cells compared to Non target (P = 0.653 ± 0.001) and OE-PEX11B cells (P = 0.693 ± 0.001), confirming enhanced fluidity (Fig 7I). In line with these results, fluorescence recovery after photobleaching (FRAP) assays—used to assess lateral membrane diffusion—using DiOC18 demonstrated accelerated recovery kinetics in KO-PEX11B cells, with a 5% higher diffusion coefficient (0.068 ± 0.025 μm^2^/s) and an increased FRAP ratio (0–40%) relative to controls (0-24.2%, 0.104 ± 0.035 μm^2^/s) (Fig 7J–7L). Additionally, the mobile fraction (Mf), an indicator of the proportion of lipids able to move freely within the membrane, exhibited significant differences between groups (*p* < 0.005, Fig 7M). Together, these findings establish PEX11B as a modulator of membrane fluidity, offering mechanistic insights into peroxisome-virus interactions.

### 2.8 Increased lipid membrane fluidity promotes ORFV infection

**Fig 8:**
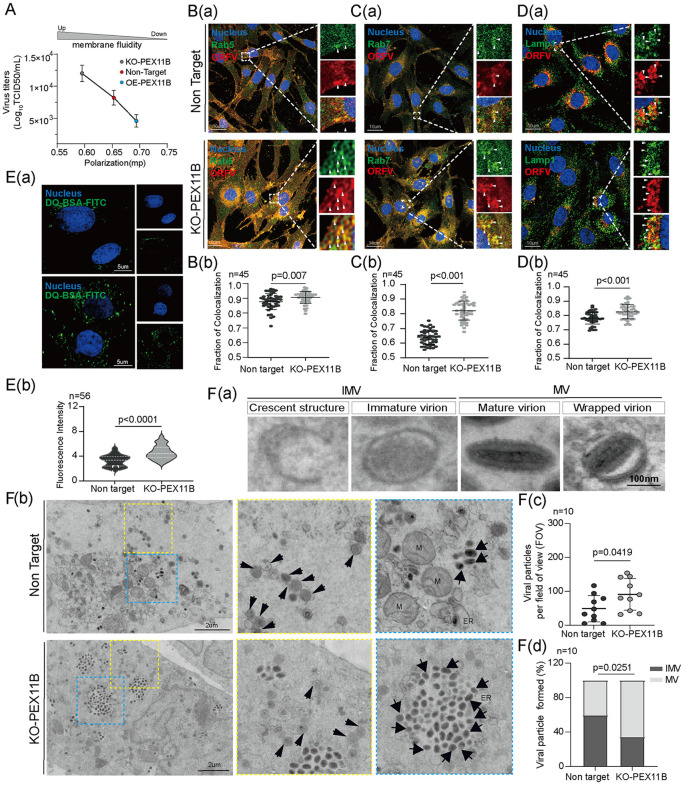
Increased lipid membrane fluidity promotes ORFV replication. (A) Cell lines with graded membrane rigidity (KO-PEX11B, Non target, and OE-PEX11B) were infected with ORFV, and the correlation between infection efficiency and TMA-DPH fluorescence polarization was analyzed. Data show an inverse correlation between viral infection efficiency and TMA-DPH fluorescence polarization. (B) (a)Confocal microscopy analysis of co-localization of ORFV and endosomes in Non target versus PEX11B knockout cell lines, 10 min (GFP-Rab5 early endosomes) after infection.Blue, Hoechst 33258; red, R18-labeled ORFV; green GFP, Rab5 early endosomes. (b) Histograms of Rab5 early endosomes and virus co-localization analysis, cell n = 45. (C) (a)Confocal microscopy analysis of co-localization of ORFV and endosomes in Non target versus KO-PEX11B cell lines, 20 min (GFP-Rab7 late endosomes) after infection.Blue, Hoechst 33258; red, R18-labeled ORFV; green GFP, Rab7 late endosomes.(b) Histograms of Rab7 late endosomes and virus co-localization analysis, cell n = 45. (D) (a)Confocal microscopy analysis of co-localization of ORFV and endosomes in Non target versus KO-PEX11B cell lines 30 min (GFP-Lamp1 lysosomes) after infection. (b) Histograms of Lamp1 lysosomes and virus co-localization analysis, cell n = 45. (E) (a)Confocal microscopy analysis of Non target, KO-PEX11B cells using DQ-green bovine serum albumin (BSA). Scale bar = 5 µm. (b)The violin plot depicts the relative fluorescence intensity for different cells. Colocalization analyses are consistent with the previous description, n = 56. (F) (a) Representative TEM images illustrating sequential stages of ORFV assembly: crescent structure, immature virion, mature virion, and wrapped virion. Scale bar = 100 nm. (b) TEM micrographs of ORFV-infected Non target and KO-PEX11B cells. Arrows indicate viral particles; yellow and blue boxes denote regions magnified in adjacent panels. Small arrow indicates immature virion; large arrow indicates mature virion.Scale bar = 2 μm. (c) Quantification of viral particles per field of view (FOV) in Non target and KO-PEX11B cells (n = 10 FOVs). Data are presented as mean ± SEM; unpaired t-test, p = 0.0419. (d) The proportion of Intracellular Mature Virions (IMV) and Mature Virions (MV) in Non target cells and KO-PEX11B cells. Unpaired t-test, p = 0.0251.

To systematically assess the impact of host membrane fluidity on viral infection, we infected isogenic cell lines (KO-PEX11B, Non target, and OE-PEX11B) with ORFV. Because these cell lines exhibited graded membrane rigidity, we quantified infection efficiency relative to TMA-DPH fluorescence polarization, a marker of reduced membrane fluidity (Fig 8A). Notably, KO-PEX11B cells with elevated fluidity supported significantly higher ORFV entry (Fig 4D), suggesting a fluidity-dependent penetration mechanism. To dissect this further, we performed time-resolved confocal imaging of R18-labeled ORFV, revealing accelerated endosomal transit in KO cells: viral particles showed enhanced Rab5 + early endosome colocalization by 10 min post-infection (Fig 8B), rapid progression to Rab7 + late endosomes by 20 min (Fig 8C), and premature Lamp1 + lysosomal accumulation by 30 min (Fig 8D). These timepoints coincide precisely with the viral uncoating phase [48–50]. Further supporting this, lysosomal activity assays confirmed heightened endolysosomal function in KO cells (Fig 8E). Collectively, these results establish membrane fluidity as a critical positive regulator of ORFV entry by accelerating vesicle maturation.

Transmission electron microscopy of KO-PEX11B and Non target cells revealed conserved stages of ORFV morphogenesis (Fig 8F(a)). Moreover, KO-PEX11B cells exhibited a marked increase in viral particle density (Fig 8F(b)). Consistent with this, quantitative morphometric analysis showed more mature, envelope-intact virions in KO-PEX11B populations (Fig 8F(c,d)). Together, these findings directly link membrane fluidity to assembly fidelity. Thus, these data establish that suppression of fluidity by PEX11B acts as an innate barrier to ORFV infection, impacting both viral entry via endosomal trafficking and late-stage virion production.

## 3 Discussion

**Fig 9:**
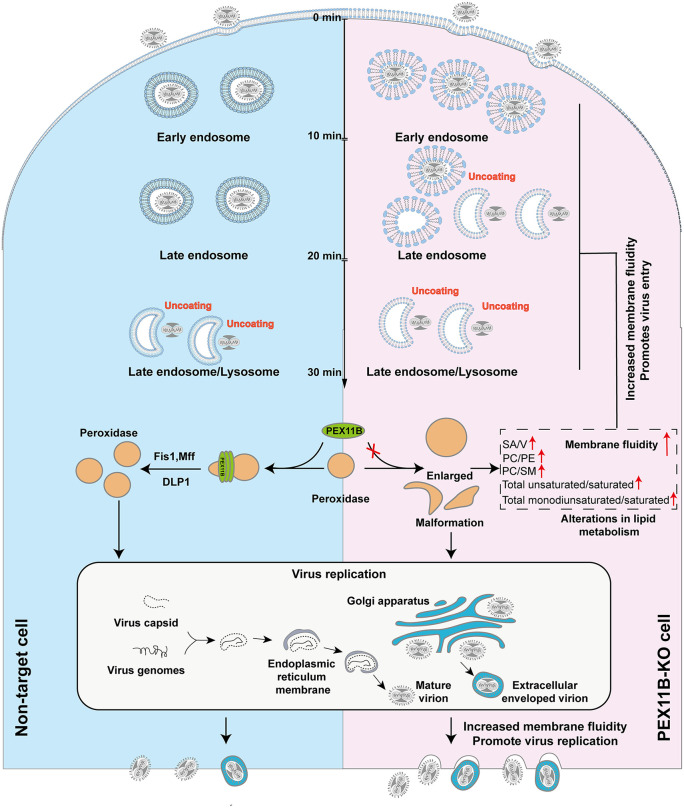
A model illustrating the roles of PEX11B in the ORFV replication cycle. This schematic outlines the role of PEX11B in regulating peroxisome dynamics and downstream ORFV infection processes. In Non target cells, PEX11B mediates proper peroxisome fission through interactions with Fis1, Mff, and DLP1, maintaining normal peroxisome morphology. In PEX11B-knockout cells, impaired peroxisome fission leads to enlarged and malformed peroxisomes, which induce alterations in lipid metabolism (e.g., changes in SA/V, PC/PE, PC/SM, and unsaturated/saturated lipid ratios). These metabolic changes first enhance lipid mobility, which in turn increases membrane fluidity and alters lipid proportions. Collectively, these effects promote key ORFV biological processes: enhanced ORFV entry (including membrane fusion), accelerated ORFV trafficking (from early to late endosomes/lysosomes with uncoating), and facilitated ORFV replication (encompassing genome replication, capsid assembly, maturation via the endoplasmic reticulum and Golgi apparatus, and release of enveloped ORFV virions). Thus, PEX11B deficiency disrupts peroxisome homeostasis, triggering a cascade of lipid-related changes that drive augmented ORFV infection.

In this study, we performed the ovine genome-wide CRISPR/Cas9 knockout screen in primary OA3.Ts cells. This allowed us to systematically identify host factors that regulate ORFV (Orf virus) infection. Our screen identified PEX11B as a critical antiviral regulator (Fig 9). When PEX11B was knocked out, ORFV replication increased. This was shown by more syncytia at 36 hpi and a 4.23-fold increase in viral titer at 48 hours. We also observed peroxisomal remodeling: fewer peroxisomes, but greater peroxisome volume and surface area. This led to a 15.7% rise in membrane-to-lumen ratio (from 23.89 to 27.64; Fig 6). These structural changes matched with higher lipid metabolic efficiency, as shown by LC-MS lipidomics (liquid chromatography-mass spectrometry), altered phosphatidylcholine/phosphatidylethanolamine and sphingomyelin ratios, increased membrane fluidity (measured by TMA-DPH and FRAP assays), and faster viral infection.

Pooled CRISPR screening enables systematic interrogation of thousands of genetic perturbations in a single experiment, but its successful implementation requires a well-characterized, optimized cellular model, with critical considerations including genetic background, growth rate, and transduction efficiency [51, 52]. Here, we addressed these challenges by generating a Cas9-hTERT-fused OA3.Ts primary cell line, which retained primary-cell characteristics while achieving immortalization and high transduction efficiency.

To ensure robust screening, we employed low MOI (~0.2) to limit cells to single sgRNA integrations and maintained 1,000x sgRNA coverage, enhancing reproducibility and sensitivity for detecting changes in sgRNA abundance [53]. Library diversity is inherently biased by sgRNA representation, necessitating large cell populations to capture underrepresented guides. Our approach optimized these parameters, ensuring reliable hit identification.

Conventional CRISPR screens for viral host factors often rely on cell survival or death as endpoints, limiting discovery to genes essential for viral replication while missing inhibitory factors. To address this, we employed ORFV antibody-coupled fluorescence to enrich a GFP-high population (top 5%), identifying gene knockouts that potentially enhanced infection. This approach revealed multiple novel ORFV-associated host factors, including RAD52, USP45, CSNK1G3, DNAH8, CDH13, IL22, LRP12, GJB6, and SLC12A5, which are worthy of further study. We discovered these genes involved in diverse biological processes, including gluconeogenesis, regulation of MAPK cascade, deoxyribonuclease I activity, DNA nuclease activity, intracellular chlorideion homeostasis, organelle membrane contact site, etc. Knockout validation confirmed the functional relevance of these candidates, supporting the screen’s reliability.

PEX11B, a peroxisomal membrane protein regulating fission by modulating membrane dynamics [42], exhibits an antiviral role whose mechanisms were previously unclear. We found that PEX11B knockout reduced peroxisome number but increased volume and surface area, yielding a 15.7% higher membrane-to-lumen ratio (Fig 6), suggesting morphological restructuring (e.g., spherical to tubular/dumbbell shapes) that expands membrane contact sites [54–56]. We further validated this change of form through pharmacological intervention experiments. Wy14643 further raised the peroxisomal surface area-to-volume (SA/V) ratio to 28.27, closely matching the morphological characteristics seen in KO-PEX11B cells, and significantly enhanced ORFV replication. In contrast, treatment with GW6471 lowered the SA/V ratio to 23.76, showing notable antiviral effects. The peroxisomal SA/V ratios in the two drug-treated groups displayed completely opposite patterns that closely corresponded with the observed viral infection outcomes.Further investigations are required to clarify the mechanisms underlying drug-mediated PEX11B inhibition and other PPARα-associated pathways. Although unresolved by high-resolution imaging, the enlarged surface area likely facilitates (1)greater transmembrane transport (e.g., fatty acid uptake for viral lipid synthesis), (2) enhanced lipid metabolic flux (supplying phospholipids for viral envelopes) [57–59], and (3) improved membrane fluidity (accelerating viral entry/egress) [60]. PEX11B deficiency also disrupted lipid homeostasis, elevating PC/PE and PC/SM ratios that are crucial for lipid raft signaling and may thereby influence viral entry, glycoprotein trafficking, or assembly. While these findings align with peroxisomal lipids’ pro-viral roles [61, 62], the exact interplay between PEX11B, membrane dynamics, and ORFV restriction requires further investigation.

Peroxisomes acquire membrane lipids from the endoplasmic reticulum (ER) and the trans-Golgi network (TGN), and their intracellular positioning is dynamically regulated in response to metabolic and pathogenic cues, which underscores the close link between peroxisomal behavior and cellular metabolic and pathogenic responses and further indicates that investigating the interplay between viral infection, TGN dynamics, and peroxisomal behavior represents a critical next step derived from the present study [63, 64]. Previous studies have described divergent functions of PEX11B and PEX3 in the regulation of plasmalogen metabolism upon viral infection [55, 65]. Unlike PEX3-deficient cells, which lack intact peroxisomes and fail to elevate plasmalogen content post infection, PEX11B knockout cells still support infection-induced plasmalogen accumulation [55]. This divergence further explains why disruption of PEX3 causes robust inhibition of virus replication: complete loss of functional peroxisomes not only damages organelle structure and metabolism, but also blocks the virus-triggered upregulation of plasmalogens. By contrast, PEX11B depletion primarily alters peroxisome quantity and morphology without ablating core peroxisomal function, so cells remain competent to modulate plasmalogen production to sustain viral propagation. As a core peroxisomal receptor protein, PEX5 dysfunction is universally exploited by diverse DNA and RNA viruses: viral proteins often trigger PEX5 ubiquitination and subsequent proteasomal degradation to eliminate peroxisome-derived innate immune signaling hubs [66, 67]. While PEX5 deficiency evidently suppresses ORFV propagation in host cells, the key post-translational modifications of PEX5 and their roles in modulating peroxisome architecture during infection require further exploration. Given that poxviruses extensively reprogram host organelle dynamics and metabolism to support their replication, PEX11B may exert its antiviral effects not only by regulating peroxisomal integrity and lipid metabolism (as shown in Fig 9) but also by modulating FIS1-dependent mitochondrial function—an effect that could potentially impact cellular redox homeostasis, bioenergetics, or innate immune signaling, which together reveals the multi-faceted involvement of peroxisomal regulation in the interplay between poxviruses and host cells [42, 68].

By establishing a genome-wide CRISPR screen in primary ovine cells, we identified PEX11B as a broad-spectrum gatekeeper of viral infection and elucidated its role in peroxisome-mediated antiviral defense. Our findings expand the understanding of peroxisomal plasticity in viral pathogenesis and highlight lipid metabolism as a critical regulatory node. The screen’s top hits, including unvalidated candidates, present promising avenues for dissecting ORFV pathogenesis and broader ovine infectious diseases. Moreover, the methodological pipeline developed here provides a scalable platform for investigating host-pathogen interactions in livestock, with potential applications in vaccine development and antiviral therapies.

## 4 Materials and methods

### 4.1 Cell culture and virus

Ovine Fetal Testis(OA3.Ts) cell lines and HEK293T were cultured in high-glucose DMEM (Gibco) with 10% fetal bovine serum, 1% MEM non-essential amino acids, 100 units/ml penicillin, and 100 mg/mL streptomycin (all Gibco). Cells were incubated at 37 °C and 5% CO_2_. The OA3.Ts cell line used in this study was kindly provided by Professor Weiye Chen of the Harbin Veterinary Research Institute, Chinese Academy of Agricultural Sciences. This cell line was originally purchased by Professor Chen from the American Type Culture Collection(ATCC:CRL-6546). The ORFV (attenuated China vaccine strain; GenBank accession no. JQ904789) was generously provided by Keshan Zhang (School of Animal Science and Technology, Foshan University).

### 4.2 Plasmids and antibodies

pMD2.G (Addagen,12259), pSPAX2 (Addagen,12260), Lenti-guide-puro (Addagen,52963), Lenti-Blast-Cas9 (Addagen,52962) were purchased from Addgene. pMD19-T Vector (Takera,6013) were purchased from Takara. PCDNA3.1, pLV3-CMV-MCS-3 × Myc-Neo, plasmid DNA was transfected into the indicated cells using Lipofectamine LTX Transfection Reagent (Thermo Fisher Scientific, 15338100).

Cas9 Polyclonal antibody(Proteintech, 26758–1-AP), TERT Polyclonal antibody(Proteintech, 27586–1-AP), GAPDH antibody(Cell Signaling Technology, 2118), α-Tublin antibody(Proteintech, 11224–1-AP), β-Actin antibody (Proteintech, 66009–1), PMP70 (Thermo, MA5–31368), HA-Tag Rabbit Monoclonal Antibody (Cell Signaling Technology, 3724) ORFV086 antibody(Antibodies-online, ABIN6774766), anti-Rab5 (Immunoway, YT5456), anti-Rab7 (Abcam, ab126712), anti-Lamp1 (Proteintech, 65051), Fluorescein (FITC)-conjugated Goat Anti-Rabbit IgG(H + L) (Proteintech, SA00003), Alexa Fluor 488-labeled Goat Anti-Mouse IgG (H + L)(Beyotime, A0428).

### 4.3 Construction pLV-cas9-hTERT plasmid

According to the hTERT gene sequence in the NCBI database (NCBI entry number: NM_198251.2), synthetic fragment A (produced by Shanghai Shenggong Technology Company) and fragment B (produced by Beijing Genomics Institution). Aim at fragment A and fragment B, using four pairs of specific primers: hTERT-F1/R1,hTERT-F2/R2,hTERT-F3/R3,hTERT-F4/R4(Sequence details are in S4 Table). Fragment A served as the template for PCR amplification, and fragments 1 (259 bp) and 2 (111 bp) (111 bp) were obtained, respectively. Fragment 3 (2104 bp) was generated through PCR amplification using fragment B and fragment one as templates. Additionally, fragment 4 (1828 bp) was obtained by PCR amplification using fragment B and fragment two as templates. The PCR amplification system is: 2 × PrimerSTAR GXL Buffer 10μL, dNTP Mixture (2.5mM) 4μL, Primer-F 0.5μm, Primer-R 0.5μm, Template 1μL (100ng), PrimerSTAR GXL DNA Polymerase 1μL, ddH2O 33μL. Amplified fragment three and fragment four and pLenticas9-Blast plasmid were digested with BamHI and then ligated using Infusion DNA ligase (Takara, 2011A). Then the conjugated product was transformed by the receptive Escherichia coli. Colony PCR screening for positive Pcas9-hTERT clones using specific primers (TF/TR). Plasmid constructs were isolated from some positive clones by Plasmid Miniprep Kit (QIAGEN, 12125) after being grown in LB/ampicillin medium. Successful cloning of these plasmid constructs was confirmed by digestion with restriction enzymes and by sequencing.

### 4.4 Construction of OA3.Ts/Cas9 cell line

To construct the immortalized OA3.Ts cell line overexpressing Cas9 (OA3.Ts/Cas9), the cas9 expression cassette was inserted into the human telomerase reverse transcriptase (hTERT) gene. The cas9 expression cassette was amplified from Lenti-Blast-Cas9 and linearised through BamHI digestion, followed by seamless cloning with the purified hTERT fragment using Infusion cloning. Subsequently, the construct was packaged into lentivirus using HEK293T cells and transfected into primary OA3.Ts cells. After 14 days of selection with blasticidin(Coolaber, CB2812) 5ug/mL, single clones overexpressing cas9 were obtained through limiting dilution and confirmed by western blot analysis for cas9 and hTERT expression. The established cell line was continuously passaged for over one hundred generations to assess its potential for immortalization.

### 4.5 Western blot

Cells were lysed on ice for 10–15 minutes using RIPA buffer (Thermo, 89900) supplemented with PMSF (Beyotime, ST506). After clarification by centrifugation (12,000 × g,10 minutes, 4°C), lysates were resolved via SDS-PAGE, electrotransferred to PVDF membranes, and probed sequentially with specific primary and HRP-conjugated secondary antibodies.

### 4.6 Indirect immunofluorescence assay

OA3.Ts primary cells and OA3.Ts/Cas9 cells were seeded into confocal culture dishes. Upon reaching 50% confluency, cells were fixed in 4% PFA(Solarbio, P1117), permeabilized, and blocked, followed by sequential incubation with primary and fluorophore-conjugated secondary antibodies. After DAPI(Thermo, D1306) nuclear staining, fluorescence images were captured on Zeiss LSM710 confocal microscopes.

### 4.7 Apoptosis assay

OA3.Ts/Primary, OA3.Ts/Cas9 and OA3.Ts/Cas9/P107 cells were seeded at 5 × 10^5^ cells per well in six well cell culture plates stained with a live-dead doublestaining dye (Abbkine, KTA0002) after 24 hours on the machine (Beckmancoulter CytoFLEX LX). Gating strategy (Annexin V + /PI− cells for early apoptosis; Annexin V + /PI+ for late apoptosis), analysis software (FlowJo v10), and how cumulative apoptotic rates were calculated (sum of early and late apoptotic populations).

### 4.8 Construction of the ovine genome-wide CRISPR/Cas9 sgRNA library

An ovine genome-wide CRISPR/Cas9 sgRNA knockout library consisting of 118,620 CRISPR sequences targeting 20,398 protein-coding genes, along with 1,000 Non target CRISPR sequences, was synthesized as 80-mer single-stranded oligos in the following format: TATATCTTGAGGAAAGGACGAAACACCG-N20-GTTTTAGAGCTAGAAATAGCAAGTTAA (N20:variable 20 bp CRISPR sequences). The oligos were then converted to double-stranded DNA (dsDNA) through PCR and cloned into SYN004-pKLV-U6gRNA(BbsI)-PGKpuro2A-mcherry by BbsI digestion and T4 ligation to make the ovine genome-wide CRISPR/Cas9 knockout sgRNA library. All CRISPR sequences were searched with an NGG protospacer adjacent motif (PAM) at the 3′ end of the exon regions of all protein-coding genes in the ovine genome (Taxonomy ID: 9940, reference genome: ARS-UI_Ramb_v2.0). Additionally, please adhere to the following guidelines. Ensure that the GC content is approximately between 40–80%. 2. Preferably, select the first exon region for cleavage sites. 3. Analyze the specificity of the sgRNA; the target sequence should have a unique match within the genome. 4. The sgRNA sequence should not contain stable secondary structures, such as hairpin structures. 5. The designed gRNA sequence (excluding the PAM) should not contain four or more consecutive thymine (T) nucleotides. 6. Consider the efficiency score predicted for the gRNA sequence. 7. The potential off-target or mismatch count for the gRNA sequence should be three or more. To expand the application of the library across different sheep breeds, the library simultaneously selected the gene sequences of three sheep breeds, namely the small tailed-Han sheep, dairy sheep, and Mongolian sheep, as references, and removed the sgRNAs that had differences among the three breeds. Specific content: We retrieved the genomic variant data of the three target sheep breeds from public databases and our in-house resequencing datasets. We then mapped all designed sgRNAs to the reference genome and cross-referenced them against the SNP profiles of the three breeds. Any sgRNA that overlapped with known SNP sites (i.e., the sgRNA targeting sequence contained one or more breed-specific SNPs) was excluded from the library.

### 4.9 Construction of the ovine genome-wide CRISPR/Cas9 sgRNA plasmid library

The PCR reaction was conducted in a Veriti 96-Well Thermal Cycler (Thermo Fisher Scientific) with 14 cycles. A total of 35 PCR reactions were performed, each containing 20 ng of oligo pool per 50 μL reaction volume. The PCR products were pooled, purified using a MinElute PCR Purification Kit (QIAGEN, 28004), and electrotransformed into Endura electrocompetent cells (Biosearch Technologies, USA). To ensure comprehensive coverage, parallel transformations were conducted, and colony numbers were recorded to achieve 1,000-fold coverage of the total number of sgRNAs in the library. Subsequently, the sgRNA plasmid library was extracted using a Plasmid Plus Maxi Kit (QIAGEN, Germany). The sgRNA cassettes from the plasmid library were amplified with PrimeSTAR GXL DNA Polymerase (Takara, China) as previously described. The PCR products were purified using a MinElute PCR Purification Kit (QIAGEN, Germany) and sequenced on an Illumina HiSeq2000 platform. The coverage and homogeneity of sgRNAs were analyzed using the MAGeCK algorithm. All primers used for constructing the sgRNA expression vector are listed in S4 Table.

### 4.10 Lentivirus production

HEK293T cells were seeded in T25 flasks (Thermo Fisher Scientific, 156367) at approximately 30% confluency 1 day prior to transfection. On the following day, when the cells reached 90–99% confluency, transfection was performed. For each T25 flask, the transfection mixture consisted of 2.4 µg of the plasmid containing the vector of interest, 2.4 µg of psPAX2 (Addgene, 12260), and 1.2 µg of pMD2.G (Addgene, 12259). This mixture was transfected using 18 µL of Lipofectamine LTX Reagent (Thermo Fisher Scientific, 15338030), 6 µL of Lipofectamine LTX PLUS Reagent (Thermo Fisher Scientific, 15338100), and 1.25 mL of Opti-MEM (Thermo Fisher Scientific, 31985070). Transfection parameters were scaled linearly with flask surface area for T75 and T225 flasks. The culture medium was replaced 6 hours post-transfection. The virus-containing supernatant was harvested 48 hours later, filtered through a 0.45-µm PVDF filter (Millipore Sigma, SLHV013SL), and concentrated by ultracentrifugation at 100,000 × g for 2 hours at 4°C when necessary. The virus supernatant was then aliquoted and stored at −80°C.

### 4.11 ORFV infection of the ovine genome-wide CRISPR/Cas9 knockout cell library and flow cytometry sorting

To generate ovine genome-wide CRISPR/Cas9 knockout cell libraries, a total of 4 × 10^8^ OA3.Ts/Cas9 cells were transduced with lentivirus of ovine genome-wide CRISPR/Cas9 knockout sgRNA library in the presence of 0.6 mg/mL polybrene (Sigma, 107689), at an MOI of 0.2. Following two days after lentiviral transduction, transduced cells were selected with 1 μg/mL puromycin (Coolaber, CP9231) for 10–14 days, while maintaining at least a coverage of 1000x. Subsequently, a library of 1.2 × 10^8^ OA3.Ts/Cas9 cells was seeded into 225 cm^2^ cell culture dishes (Thermo Scientific, 130330) and infected with ORFV at an MOI of 2. At 48 hours post-infection, cells were collected, fixed and permeabilized using the reagent from Cytofix/Cytoperm Fixation/Permeabilisation Kit (BD Biosciences, 554714). After washing three times with Perm/Wash Buffer (BD Biosciences, 554723), cells were incubated with ORFV antibody(Antibodies-online, ABIN6774766) for 1 hour, followed by another three washes with Wash Buffer. Cells were then incubated with Alexa Fluor 488-labelled Goat Anti-Mouse IgG(H + L) (Beyotime, A0428) for 30 minutes and washed three times with Wash Buffer. Finally, cells were resuspended in PBS containing 1% BSA and subjected to cell sorting to collect the top 5% of GFP-expressing cells. Flow cytometry was performed using a Sony MA900 FACS Flow Cytometry System and MA900 Cell Sorter software.

### 4.12 Screen analysis

We extracted genomic DNA from surviving cells using the Quick-DNA Miniprep Plus Kit (ZYMO RESEARCH, D4068). The gRNA region was amplified by PCR employing Takara Ex Taq polymerase (Takara, RR001Q) and a pair of barcoded P7/P5 primers. Following purification with the FastPure Gel DNA Extraction Mini Kit (Vazyme, DC301–01), the products were delivered to Annoroad Gene Technology (Beijing) for next-generation sequencing. Gene enrichment analysis was subsequently conducted with MAGeCk (version 0.5.9).

### 4.13 Cell viability assay

The cells were seeded into opaque-walled 96-well plates at a density of 5  × 10^3^ cells per well in 100 µL of DMEM and incubated for 24 hours. The CCK-8 assay (Dojindo, CK04) was performed to analyze cell viability at 0, 24, 48, 72, and 96 hours. The cells were incubated with the CCK-8 reaction solution for 2 hours at 37 °C. A SpectraMax Mini microplate reader (Molecular Devices, USA) was employed to measure the absorbance at 450 nm, and the optical density (OD) values were obtained to assess the cells’ proliferation abilities.

### 4.14 Generation of PEX11B knockout OA3.Ts/Cas9 single-cell clone

The construction of the lentivirus sgRNA expression vector was initiated by digesting the lenti-pcg 2.0 vector with the BbsI restriction enzyme. The paired oligonucleotides of sgRNA were annealed and subsequently cloned into the linearised vector. The oligonucleotides (PEX11B oligo F: ACCGACAGAAACAGATTCGACAAC and PEX11B oligo R: AAACGTTGTCGAATCTGTTTCTGT) were synthesized (Beijing Genomics Institution) and annealed in a 50 μL reaction containing TransTaq HiFi Buffer II at a final concentration of 9 μM. The annealed oligonucleotides were ligated into a lentiviral vector carrying a puromycin selection marker using Golden Gate Assembly (NEB). The ligation products were then transformed into Trans1-T1 competent cells (Transgen, CD501). Pseudotyped lentiviral vectors expressing sgRNA were generated by following the previously described lentivirus production steps. The OA3.Ts/Cas9 cell line was transduced with these pseudotyped lentiviral vectors and selected with puromycin (1 mg/mL). Single-cell clones resistant to puromycin were selected. Genomic DNA was extracted from these clones, and the insertions and deletions (indels) caused by sgRNA/Cas9 in each cell clone were confirmed by Sanger sequencing after PCR amplification. The monoclonal cells that stably expressed the Cas9 gene and exhibited the highest knockout efficiency were obtained through Western blot and knockout efficiency detection (TIDE: http://tide.nki.nl/).

### 4.15 Generation of PEX11B over-expression and mutant OA3.Ts/Cas9 cell line

To construct the over-expression vector for the generation of PEX11B stable cell lines, the coding sequences of PEX11B were cloned into the pLV3-CMV-MCS-3 × Myc-Neo vector (Clontech), which was linearised with BamHI. Subsequently, to abolish cleavage by sgRNA and Cas9 in KO-PEX11B cells, a specific point mutation in protospacer adjacent motif sequences, which does not alter the amino acid, was introduced into the PEX11B coding sequences to construct its mutant over-expression vectors. The PEX11B mutant was generated following the same procedure as described above. Sanger sequencing was performed to confirm all plasmids. All primer sequences are listed in S4 Table.

### 4.16 RNA isolation and RT-qPCR

The cells were lysed in TRIzol (Tiangen, DP424), and RNA was isolated according to the manufacturer’s protocol. The cDNA was synthesized using the Evo M-MLV RT Premix for qPCR (Accurate Biology, 11728). RT-qPCR was performed using TB Green Premix Ex Taq II FAST qPCR (Takara, CN830A). Gene expression was quantified using the 7500 Real-Time PCR System (Thermo Fisher Scientific). The sequences of the primers targeting ORFV B2L were as follows: GGGCTCTACTCCACCAACAA (forward) and CGAGTCCGAGAAGAATACGC (reverse). The expression of target genes was normalisednormalized to β-actin were as follows: CCATCGTCCACCGCAAAT (forward) and CAAATAAAGCCATGCCAATCTC (reverse).

### 4.17 ORFV infection and purification

OA3.Ts/Cas9 cells were infected with ORFV at an MOI of 2 in serum-free DMEM, using 250 μL per well for 6-well plates or 2 mL per T75 flask. Following a 1.5 hours adsorption period at 37°C, the inoculum was removed, cells were washed, and fresh serum-free DMEM was added. After 48 hours, the supernatant was collected and cells were lysed by three freeze-thaw cycles to release intracellular viruses. The combined supernatant was clarified by centrifugation at 3,000 × g for 10 minutes and filtered through a 0.45 μm membrane. Viral titers were subsequently determined by TCID_50_ assay. For virus concentration, the clarified supernatant was processed using the Universal Virus Concentration Kit (Beyotime, C2901S) according to the manufacturer’s protocol. Briefly, the pre-cooled Virus Precipitation Reagent was then mixed in proportion with the viral supernatant, thoroughly mixed, and stirred at a low speed at the appropriate temperature overnight. This was followed by centrifugation to remove the supernatant and careful aspiration of the precipitate. The process was then repeated, and the supernatant was collected as concentrated virus.

### 4.18 R18 loading of ORFV particles

A volume of 100 µL of purified ORFV was added to 2 µL of 5 µmol Octadecyl Rhodamine B Chloride (R18; MKBio, 65603-19-2) in phosphate-buffered saline (PBS, Quality Biological) + 0.2% bovine serum albumin (Thermo, 10010023) for 60 min at room temperature in the dark. Non-incorporated R18 was removed by a 0.45 μm sterile filter (MerckMillipore, SLHVR33RB).

### 4.19 R18-ORFV binding and entry assay

Cells were seeded on a 40 mm confocal dish(Thermofisher, 150680) and inoculated with R18-ORFV at an MOI of 100 for 60 minutes at 4°C. Cells were then washed twice with ice-cold PBS to remove unbound virus and fixed with 4% paraformaldehyde (Beyotime, P0099). Following a 10 minutes incubation with hochest 33258 (Thermo, H1398) staining solution at room temperature, the cells were washed three times with PBS and subsequently imaged with a confocal microscope. Entry assay: Cells were subjected to adsorption at 4°C and washed with pre-chilled PBS to remove free virions, followed by incubation at 37°C for 1.5 hours. After incubation, the cell surface was treated three times with pH 3 PBS to completely eliminate uninternalized residual virions. Subsequently, DNA is extracted for quantitative qPCR experiments.

### 4.20 Acid-bypass experiment

Cells were incubated with ORFV at 4°C for 1 hour to allow viral adsorption. After incubation, the cells were washed three times with pre-chilled PBS to remove unbound free virions. Then cells were treated with citrate buffer (pH 5.0) at 37°C for 2 minutes. Artificial acidification was used to trigger viral membrane fusion (mimicking the endosomal acidification process), thus enabling direct viral membrane fusion independent of the endocytic pathway. Immediately after the acid treatment, the system pH was neutralized with complete medium, and the cells were washed twice with PBS to eliminate residual acid buffer. The cells were incubated at 37°C for 10 hours, then harvested for intracellular viral RNA extraction. The viral genome copy number was quantified via qPCR assay.

### 4.21 Measuring lysosomal degradation of DQ-BSA

A total of 1 × 10^5^ OA3.Ts/Cas9 cells were seeded on a 40 mm confocal dish (Thermofisher, 150680) and incubated with 10 μg/mL DQ Green BSA (Sharebio, D-12050SB). Following 8 hours of standard culture, the dye-containing culture medium was subsequently replaced. 200 μL DAPI for one hour at 37°C. The cells were then washed in PBS and fixed in 4% paraformaldehyde. Confocal microscopy Images (Zeiss LSM 710 Confocal Microscope) were acquired with a × 40 objective.

### 4.22 TCID_50_ assay

OA3.Ts/Cas9 cells were seeded at a density of 1 × 10^5^ cells per well in 96-well plates and cultured for 24 hours at 37°C in 5% CO_2_. The ORFV stock was thawed on ice and serially diluted 10-fold in DMEM containing 2% serum, typically preparing 6–8 dilutions with 8 replicate wells per dilution. The culture medium was aspirated, and cells were washed three times with PBS. Then, 100 μL of each virus dilution was added to the corresponding wells. The plates were incubated at 37°C in 5% CO_2_ for 1 hour, with gentle shaking every 15 minutes to ensure uniform virus distribution. After incubation, the virus inoculum was aspirated, cells were washed three times with PBS, and 200 μL of maintenance medium (DMEM with 2% serum) was added to each well. The plates were returned to the incubator and monitored daily under an inverted microscope for the appearance of cytopathic effect (CPE). The final reading was performed on day 5 post-infection. The number of CPE-positive wells per dilution was recorded, and the TCID_50_ was calculated using the Reed-Muench method.

### 4.23 LC-MS

The mass spectrometry signal acquisition of the sample was performed in separate positive and negative ion modes. The specific acquisition mode employed was data-dependent acquisition (DDA). Non target cells and KO-PEX11B Cells were transferred into a 2 mL centrifuge tube with 600 μL methanol-water (V: V = 1:1). An internal standard (Lyso PC-17:0, 0.1 mg/mL, prepared in methanol) was added at a volume of 20 μL. Subsequently, 600 μL chloroform was added to the mixture. The sample was subjected to sonication in an ice bath at 500 W for 3 minutes with a cycle of 6 second on and 4 second off. After that, the sample was subjected to ultrasonic extraction in an ice-water bath for 10 minutes. The mixture was then left to stand at 4°C for 30 minutes and centrifuged at 12000 rpm for 10 minutes at 4°C. The lower phase (400 μL) was collected and transferred to an LC-MS vial for evaporation. In the remaining centrifuge tube, 600 μL chloroform-methanol (V: V = 2:1) was added. The mixture was vortexed for 30 second and subjected to ultrasonic extraction in an ice-water bath for 10 minutes. The mixture was left to stand at 4°C for 30 minutes and then centrifuged at 12000 rpm for 10 minutes at 4°C. The lower phase (400 μL) was collected and transferred to the same LC-MS vial for further evaporation. The lipid residue in the LC-MS vial was re-dissolved in 300 μL isopropanol-methanol (V: V = 1:1) by vortexing for 30 second and sonicating in an ice-water bath for 3 minutes. The solution was then transferred to a 1.5 mL EP tube and centrifuged at 12000 rpm for 10 minutes at 4°C. The supernatant (200 μL) was transferred to an LC-MS vial with an insert for LC-MS analysis of Ultra-High Performance Liquid Chromatography Tandem High-Resolution Mass Spectrometer (Dionex U3000 UHPLC; UHPLC-HRMS/MS). The quality control (QC) sample was prepared by mixing equal volumes of the extraction solutions from all samples, with each QC sample having the same volume as the individual samples. Note: All extraction solvents were pre-cooled at -20°C before use.

### 4.24 Fluorescence recovery after photobleaching (FRAP) assay

For FRAP experiments, cells have been seeded in a 40 mm confocal dish (Thermofisher, 150680) with 2 × 10^5^ cells per dish the day before the experiment. The solution has been removed completely, and 100 μL of DiOC18 (MKBio, MX4001) in phosphate-buffered solution has been added. After 15 min of incubation at 37˚C, cells were washed three times with PBS. FRAP measurements were performed on a ZISSE LSM710 confocal microscope using a 40x water objective. The incubation chamber around the microscope was set to 37˚C. Imaging was performed with the 488 nm laser line of an argon laser set to 70% laser power with less than 10% transmission. Images were acquired at a rate of 1.318 s/image. Five frames were recorded before bleaching and 72–100 after bleaching. Bleaching was performed by zooming in on the bleaching area and scanning once with all lines of the argon laser at 100% transmission, plus a UV diode at 100% transmission. This resulted in bleaching of > 80% of the fluorescence intensity in the bleached spot. For analysis, the background fluorescence outside of the cells was first subtracted from the image. The intensity in the bleached spot was normalized to the intensity in the whole cell to correct for the total loss of fluorescence during the experiment. It was further normalized to pre-bleaching intensity in the spot.

The mobile fraction (*mf*) was calculated using the asymptote (*y*_*0*_) of this function:

*mf* = *(y*_*0*_ - *I*_*bleached*_ */ I*_*prebleached*_*-I*_*bleached*_*)*

Where Ibleached is the fluorescence intensity in the bleached area in the first frame after bleaching, and Iprebleached is the mean intensity in that area in the five frames before bleaching. Accordingly, the immobile fraction (*If*) is:

*If=1-mf*

The diffusion constant (*D*) was calculated using the time to reach half maximum (τ_1/2_) of the exponential fit and the equation for two-dimensional diffusion:

*D*=R^2^/4τ_1/2_

### 4.25 Fluorescence anisotropy evaluation

The fluorescence anisotropy of 1-[4-(trimethylamino)phenyl]-6-phenyl-1,3,5-hexatriene (TMA-DPH, MCE, HY-D0986) incorporated in the cells was assessed by the determination of steady state fluorescence polarisation of the membrane-fluorescent probe system; the TMA-DPH probe lacks fluorescence in solution and becomes fluorescent when incorporated into the lipid membrane bilayer. 1 × 10^5^ cells were collected and suspended in a solution containing one µM TMA-DPH. The suspension was incubated at 4°C for 20 minutes, followed by centrifugation at 800 rpm for 3 minutes. The supernatant was discarded, and the cells were resuspended in 2 mL of PBS (pH 7.4). The cell suspension was then aliquoted at 150 µL per well into a black microplate for detection with a Spectra Max iD5 Microplate detection instrument with blue polarised light detection (excitation wavelength 355 nm; emission maximum 430 nm) at room temperature. Calculation of the fluorescence anisotropy (P) was performed according to equations (A) and (B):

(A)R=(*I*_VV_*-GI*_V0_)/(*I*_VV+_*GI*_V0_)

(B)G = *I*_0V_/*I*_00_

Where P is the fluorescence anisotropy, I_vv_, I_vo_, I_ov_ and I_oo_ represent the emission intensity corrected for the autofluorescence signal of unstained cells, when the polarisers in the excitation end emission beams are oriented in vertical-vertical, vertical-horizontal, horizontal-vertical and horizontal-horizontal positions, respectively.

### 4.26 Confocal microscopy

For 100uL R18-ORFV with 900 µL of PBS, and incubated under light-avoiding room temperature conditions for 1 hour. At the conclusion of the labelling process, any unbound R18 was filtered out using a 0.45 μm filter. Cells were subjected to three washes with PBS, after which the labelled virus was added and allowed to bind at 4°C for 1 hour. At the conclusion of the procedure, any unbound virus was removed by washing with PBS, and the cells were incubated at 37°C for the indicated times to allow for internalization (early endosomes: 10 minutes, late endosomes: 20 minutes, lysosomes: 30 minutes). The cells were fixed using 4% paraformaldehyde for a period of 10 minutes. Subsequently, the cells were punched using Immunostaining Permeabilisation Buffer with Saponin (Beyotime, P0095), in accordance with the instructions provided by the manufacturer. The second antibodies were used in a 1:300 configuration: anti-Rab5 (Immunoway, YT5456), Rab7 (Abcam, ab126712), and Lamp1 (Proteintech, 65051). Cells were incubated overnight. Following the application of the anti-rabbit secondary antibody (Proteintech, SA00003) in a 1:200 configuration, the cells were incubated for a period of 2 hours. Subsequently, the cells were stained with Hoechst 33258 Staining Solution for 10 minutes. The images were captured using a Zeiss LSM 710 equipped with a 20x Plan-Apochromat objective with NA = 0.8 and a 63x PlanApo oilimmersion objective with NA = 1.4, operated with the ZEN 3.8 imaging software (Zeiss). The following laser lines and detection windows were used for excitation and emission: Hoechst 33258(nucleus): Excitation with a 405 nm diode laser, emission collected through a 420–480 nm band-pass filter; Alexa Fluor 488 (for Rab5, Rab7, Lamp1): Excitation with a 488 nm argon laser, emission collected through a 500–550 nm band-pass filter;R18 (labeled virus): Excitation with a 543 nm helium-neon laser, emission collected through a 560–615 nm band-pass filter.

## 4.27 3D reconstructions

All peroxisome-related microscopy work was performed using a Nikon AXR confocal microscope system. This integrated system incorporates a high-speed resonant scanning confocal unit, a high-sensitivity sCMOS camera, and a high-precision motorised stage optimised for advanced imaging applications. Images (1024 × 1024 pixels) were captured on a Nikon AXR confocal microscope using a 100 × oil objective with a 1.4 numerical aperture. Z-steps of 0.2 microns were used for analysis of peroxisome morphology and colocalization. FIJI (Fiji Is Just ImageJ,V2.17.0)software was used to classify surfaces, eliminate noise in each channel, and create 3D reconstructions.

### 4.28 Gene editing efficiency testing by T7E1 digestion and TIDE analysis

Seven to eight days after transfection with lentivirus or individual CRISPR sgRNAs, crude genomic DNA was extracted from cells using QuickExtract DNA Extraction Solution following the product manual. The extract was diluted 1:10 in nuclease-free water, and 1 μL of the dilution was used for PCR using primers flanking the target site and Phusion HF polymerase with supplied buffer, producing amplicons between 300–800 bp in size. 2 μL of the PCR reaction was run on 2% agarose gel to estimate the amplicon concentration. Without purification, the calculated volume of PCR reaction containing ~200ng PCR product based on the agarose gel was denatured at 95°C for 5 minutes and re-annealed by dropping the temperature to 25°C at 0.1°C per second in a thermal cycler. At the end of the program, the temperature was reduced to 4°C. Right after re-annealing, one μL T7 Endonuclease I was added to the PCR reaction directly and incubated at 37°C for 30 minutes. Immediately after incubation, the reaction was analysed on a 2% agarose gel. The Densitometry function of ImageJ processed the gel image, and the percentage editing was calculated. The editing efficiency was also examined by TIDE analysis (https://tide.deskgen.com/)following Sanger sequencing of purified PCR products.

### 4.29 Detection of IMV (immature virions) and MV (mature virions) during ORFV infection using transmission electron microscopy

The process of ORFV infection was observed using transmission electron microscopy. Briefly, Non target cells and KO-PEX11B cells were infected with viruses (MOI = 1) and incubated at 4 °C for 1 hour. After incubation, PBS was used to wash away unbound viruses. The bound virus was then allowed to infect at 37 °C for 48 hours. Following infection, the cells were fixed with 2.5% glutaraldehyde for 16 hours. Finally, ultrathin sections were prepared on carbon-coated 100-mesh copper grids and observed in a Hitachi-7650 transmission electron microscope at an operating voltage of 80 kV.

### 4.30 Statistical analysis

Statistical analyses were conducted using GRAPHPAD Prism 9.0 software(La Jolla, CA, USA). All experiments were repeated at least three times, and the distribution of data points is presented as mean SEM. Results are shown as means ± standard deviations (SD) of the data obtained from three independent experiments or triplicates as indicated. Comparisons between samples were done using the paired two-tailed t-test. *p* < 0.05 was considered significant.

## Supporting information

S1 Fig Validation of the construction of pLV-cas9-hTERT plasmid (Related to Fig 1). (A) Restriction enzyme digestion identification of recombinant pLV-Cas9-hTERT plasmid. M: DL15,000 DNA molecular weight marker; Lane 1: pLV-Cas9-hTERT digested with BsmBI (329 bp, 1994 bp and 14064 bp fragments); Lane 2: pLV-Cas9-hTERT linearized by single EcoRI digestion (single 16387 bp band); Lane 3: pLV-Cas9-hTERT double-digested with BsmBI and EcoRI (329 bp, 1994 bp, 1645 bp and 12419 bp fragments); Lane 4: undigested intact pLV-Cas9-hTERT plasmid. (B) Sanger sequencing verification of recombinant pLV-Cas9-hTERT plasmid. Representative sequencing chromatograms of the inserted fusion fragment from pLV-Cas9-hTERT construct. Regions highlighted by boxes correspond to the sequencing reads spanning the recombinant ligation junctions of the target insertion.
(TIF)

S2 Fig Verification of immortalized OA3.Ts/Cas9 cells (Related to Fig 1). (A) eGFP expression in OA3.Ts primary cells transduced with different concentrations of lentivirus. Primary OA3.Ts cells were infected with eGFP-encoding lentivirus at either 1× (undiluted lentiviral stock) or 4×(four-fold concentrated lentiviral stock). Scale bar = 100 μm. (B)PCR detection of Cas9-hTERT fragment in OA3.Ts/Cas9 cells at different passage numbers. Genomic DNA was extracted from OA3.Ts/Cas9 cells at indicated passage numbers (P07, P27, P47, P67, P87, P107). Mock, untreated primary cells. (C) Morphological comparison between primary OA3.Ts cells and OA3.Ts/Cas9 cells at late passage. Representative phase-contrast (or brightfield) images showing the morphology of primary OA3.Ts cells and OA3.Ts/Cas9 cells at passage 107 (P107). Scale bar = 500 μm. (D) Proliferation curves of OA3.Ts primary cells and OA3.Ts/Cas9 cells at different passage numbers. (E) Detection of apoptosis efficiency in cells of different passage generations(a)Summary chart of apoptosis efficiency under different passage numbers.(b)Flow cytometry plots of apoptosis efficiency at different cell passage numbers.Vertical axis (PE-H): Annexin V-PE fluorescence, indicating phosphatidylserine externalization (early apoptosis).Horizontal axis (Comp-FL2-H): PI fluorescence, reflecting loss of membrane integrity (late apoptosis/necrosis).Quadrant analysis:Q1 (upper left): Necrotic or late apoptotic cells (Annexin V ^−^ / PI⁺).Q2 (upper right): Late apoptotic cells (Annexin V ⁺ / PI⁺).Q3 (lower right): Early apoptotic cells (Annexin V ⁺ / PI^−^).Q4 (lower left): Viable cells (Annexin V ^−^ / PI^−^).
(TIF)

S3 Fig Establishment of a monoclonal Cas9-expressing cell line by single-cell cloning (Related to Fig 1). (A) Cas9 mRNA expression levels in OA3.Ts/Cas9 clonal cell lines measured by qPCR. Total RNA was extracted from seven OA3.Ts/Cas9 clonal cell lines (Clone A through Clone G), and reverse-transcribed to cDNA. Cas9 gene expression was quantified by quantitative real-time PCR (qPCR) and normalized to the housekeeping gene β-actin. Each bar represents mean ± SEM (or SD) from at least three technical replicates. (B) hTERT mRNA expression levels in OA3.Ts/Cas9 clonal cell lines measured by qPCR. (C) Evaluation of Cas9 activity among candidate single-cell-derived clones using a T7EN I cleavage assay. The candidate cells were transduced with a validated sgRNA (targeting the B4GALNT2 gene) lentivirus. The single-cell-derived clone with the highest Cas9 activity is Clone#A. Indels% indicates the percentage of alleles with insertions or deletions (indels) at the target locus, determined by T7EI mismatch cleavage assay. (D) TIDE analysis of indel efficiency in monoclonal cells derived from CRISPR/Cas9-edited OA3.Ts cells. (a) Bar graph showing the percentage of monoclonal cells with knockout efficiency in seven isolated clones (Clone-A to Clone-G) as determined by TIDE analysis. (b-h) Indel spectra of individual clones analyzed by TIDE. Red bars represent insertion/deletion (indel) frequencies; the total editing efficiency and goodness-of-fit (R^2^) for each clone are indicated. Statistical significance of indel events is denoted by color (p < 0.001 vs. control).
(TIF)

S4 Fig Evaluation of the cleavage efficiency of Cas9 in OA3.Ts/Cas9/clone#A cells (Related to Fig 1). (A) Cas9 and hTERT expression does not affect ORFV infection in OA3.Ts/cas9 clone. ORFV virus titers in parental OA3.Ts primary cells and three OA3.Ts/Cas9 monoclonal clones (Clone-A, Clone-B, Clone-C) were determined by TCID_50_ assay at 24h post-infection with an MOI of 0.001. (B) Assessment of the infection efficiency of sgRNA lentivirus in OA3.Ts/cas9/clone#A cells at 24h post-infection with an MOI of 0.001. (C) Assessment of gene editing efficiency in OA3.Ts/Cas9/clone#A cells using the T7E1 endonuclease digestion assay. Results indicate that the gene editing efficiency tends to stabilize at 6 days following lentiviral infection. Scale bar,100um. (D) Assessment of the cleavage activity of sgRNA lentivirus in OA3.Ts/cas9/clone#A cells at the time points indicated using a T7EN I assay. Indel%: percentage of indels; bp: base pairs; dpi: days post infection, Control: wild-type cells; Marker: Marker I DNA ladder.
(TIF)

S5 Fig Generation of ovine genome-wide sgRNA cell libraries (Related to Fig 2). (A) Overview table of distribution of ovine genome-wide sgRNA Library. The library comprises a total of 119,620 targeting guide RNAs (sgRNAs) directed against 20,398 protein-coding genes. Among these, 19,312 genes are targeted by 6 guides per gene, while the remaining genes are covered by 5–1 guide(s) (131, 163, 200, 249, and 343 genes for 5, 4, 3, 2, and 1 guide(s), respectively). Additionally, 1,000 non-targeting control guides are included. The design ensures broad and balanced coverage for genome-scale functional screening applications. (B) The count of mismatches for each sgRNA in the library. The information is derived from the CRISPR sgRNA library of the sheep whole genome, which is utilized to evaluate the targeting precision and off-target potential of sgRNAs. (C) Sequencing read distribution accumulation of the sgRNA plasmid library. The majority of sgRNAs exhibit read counts between 1.5 and 3.5 log_10_, with a peak around 3.0-3.5, indicating balanced library representation. (D) Evaluation of knockout effects of a randomly selected sgRNA (UVRAG,LIPC,S100A3) from the initially designed sgRNA library by T7EI assay. (E) Analysis of the positive rate of cells following lentiviral infection (with 5-fold serial dilution) using flow cytometry.
(TIF)

S6 Fig Identification of the enhanced viral potential in clonal RAD52, USP45, CDH13, LRP12, PEX11B, GJB6, and SLC12A5 knockout cells (Related to Fig 3). (A) CRISPR knockout efficiency analysis of candidate genes.(a) Bar graph showing the percentage of gene knockout in NEHJ and unmodified groups for the top candidate genes identified in the CRISPR screen.(b) Indel spectra of each candidate gene analyzed by TIDE. Red bars represent the frequency of insertion/deletion (indel) events, with total editing efficiency and goodness-of-fit (R^2^) indicated for each target. Statistical significance of indel frequencies is denoted by color (p < 0.001 vs. control). (B) The CPE (cytopathic effect) of seven-knockout cell lines infected at different time points. (MOI = 2) Scale bar = 100 μm. (C) (a) Representative immunofluorescence images of seven-knockout cell lines infected with ORFV (MOI = 2) for 48 h. Scale bar = 50 μm. (b)The normalized average intensity of ORFV V086 signal per cell, with the Non target control set to 1. Data are representative of three independent biological replicates. (D) Flow cytometric analysis of ORFV086 expression in seven ORFV-infected knockout polyclonal cell and Non target cell lines related to Fig 3H.
(TIF)

S7 Fig Validation of PEX11B as a host restriction factor against ORFV infection(Related to Fig 4 and Fig 5). (A) Western blot assay to detect the PEX11B protein expressed in KO-PEX11B and Non Target cells. α-Tublin used as an internal control gene. (B) Relative quantitative real-time PCR detection of PEX11B expression levels in KO-PEX11B and Non Target cells. (C) Cell viability in KO-PEX11B versus Non target cells by cell counting kit-8 assay. (D) Table listing the top seven predicted off-target sites (OFT1-OFT7) with up to 3 mismatches. Genomic location, number of mismatches, and sequences (with mismatches highlighted in red) are shown. (E) T7 endonuclease I (T7EI) assay of the seven predicted off-target sites. No cleavage products indicative of off-target editing were detected in the KO-PEX11B cell line, confirming the absence of detectable off-target mutations. WT, wild-type. (F) Quantitative analysis of ORFV binding was performed by relative quantification targeting the early ORFV gene ORF035. (G) Elution efficiency was detected using pH = 3.0 PBS. Data are presented as means ± SD (n = 3). (H) Western blot analysis of Myc-tagged PEX11B truncation mutants.Expression of Myc-tagged PEX11B truncation mutants (Δ210–259, Δ159–183, and Δ185–201) was detected by immunoblotting using an anti-Myc antibody. α-Tublin was used as a loading control. The Myc-tagged proteins migrated at the expected size of ~29 kDa, and α-Tublin at ~50 kDa, confirming successful expression of each truncation construct.
(TIF)

S8 Fig The effects of PEX3 and PEX5 on ORFV infection. (A) Schematic illustration of peroxisome formation: PEX3 mediates membrane assembly, PEX5 mediates matrix protein import, and PEX11B controls organelle fission. (B) TIDE analysis of CRISPR-edited cells to validate PEX3 (a) and PEX5 (b) knockout efficiency. X-axis represents indel size (deletions < 0, insertions > 0); Y-axis shows the proportion of sequencing reads for each indel. Total editing efficiency and goodness-of-fit (R^2^) are displayed for each sample; significant indels (p < 0.001) are colored red. (C) Quantitative RT-PCR analysis validates efficient knockdown of PEX3 and PEX5 in respective knockout cell lines. Relative mRNA abundance of PEX3 in PEX3-knockout cells and PEX5 in PEX5-knockout cells was normalized to nontarget control cells (dashed line set to 1). (D) Relative ORFV infection levels in KO-PEX3, KO-PEX5, and KO-PEX11B cells normalized to Non Target cells. p-values reflect statistical comparisons versus control.
(TIF)

S9 Fig PEX11B ablation elicits peroxisome remodeling in morphology and size (Related to Fig 6). (A) Maximum projections of Non target and KO-PEX11B cells with anti-PMP70 and imaged at 100X. A square frame indicates ROIs in the corresponding color. Scale bars = 10 µm. (B) Plot of the surface area and volume of individual peroxisomes in (a) Non Target Mock cells (mean SA/V = 24.83 μm^-1^), (b) Non target 72hpi cells (mean SA/V = 31.83 μm^-1^),(c) KO-PEX11B (mean SA/V = 27.34 μm^-1^),(d) KO-PEX11B 72hpi cells (mean SA/V = 32.86 μm^-1^). Solid line indicates the regression curve, dashed lines indicate the upper and lower 95% confidence intervals.
(TIF)

S10 Fig PEX11B deletion reprograms the lipidomic landscape of viral and cellular membranes(Related to Fig 6). (A) Cell viability of Non target cells treated with GW6471 was assessed using the Cell Counting Kit-8. (B,C) ORFV infectious B2L gene mRNA expression level and virus titer produced from infected OA3.Ts/Cas9 cells following GW6471 treatment to inhibit peroxisome biogenesis. (D) Cell viability of Non target cells treated with Wy14643 was assessed using the Cell Counting Kit-8. (E,F) ORFV infectious B2L gene mRNA expression level and virus titer produced from infected OA3.Ts/Cas9 cells following Wy14643 treatment to induce peroxisome biogenesis. (G) Quantification of peroxisome number per cell under different group. Peroxisomes were counted via immunofluorescence staining of peroxisomal membrane marker PMP70 in Non target cells,Non target cells(vehicle DMSO), KO-PEX11B cells, Non target cells with GW6471 10 μM and Non target cells with Wy14643 25 μM. (H,I) The average volume of peroxisomes per cell is shown in (H), the average surface area of peroxisomes per cell is shown in (I). N = 22 cells in Non Target Mock, N = 22 cells in Non Target DMSO,N = 27 in KO-PEX11B Mock, N = 22 in Non Target with GW6471 10 μM, N = 24 in Non Target with Wy14643 25 μM. (J) Schematic representation of peroxisome morphological changes induced by different manipulations. Peroxisome surface area-to-volume (SA/V) ratios and representative shapes are shown for Non target cells, GW6471-treated cells (10 μM), Wy14643-treated cells (25 μM), and KO-PEX11B cells.
(TIF)

S11 Fig PEX11B deletion reprograms the lipidomic landscape of viral and cellular membranes (Related to Fig 7). (A) Statistical summary of lipidomics categorization of lipids in cells. Lipid relative abundances were quantified based on peak area normalization. Each segment represents the percentage contribution of an individual lipid class: FA (fatty acids, 4.9%), GL (glycerolipids, 21.13%), GP (glycerophospholipids, 59.14%), SL (sphingolipids, 3.96%), SP (sterol lipids, 10.45%), and ST (steroids, 0.42%). (B) Principal component analysis (PCA) of lipid profiles distinguishes Non Target and KO-PEX11B cells. PCA score plot based on global lipidomic data showing clear separation between the Non Target group (blue dots) and KO-PEX11B group (orange dots). Ellipses denote the 95% confidence interval for each group. (C) Permutation test (n = 200) validating the OPLS-DA model. The plot displays R^2^ (green triangle) and Q^2^ (blue square) values obtained after 200 random permutations of the Y matrix. The original model’s R^2^ = 0.997 and Q^2^ = -0.055 (red symbols) fall outside the permutation distribution, indicating that the observed separation is not due to over-fitting and the model possesses robust predictive power. (D)Differential lipid distribution volcano plot. Red dots represent metabolites that are upregulated in the experimental group, blue dots represent downregulated metabolites, and gray dots represent metabolites that are not significant. The horizontal axis shows the log2(FC) values for the comparison between the two groups, while the vertical axis represents -log10(p-value) values. Individual significant differences in lipids have been annotated. (E) A classified bubble chart showing the relationship between carbon chain length and unsaturation in PC(a), PE (b), and SM(c). The x-axis represents the carbon chain length of the lipids, while the y-axis represents the unsaturation of the lipids. The color of the bubbles maps to Log2(FC), and larger circles indicate smaller *p*-value values.
(TIF)

S1 File Raw images. Uncropped original western blot images corresponding to Fig 1B, Fig 4J, Fig S7A and Fig S7H.
(PDF)

S1 Table MAGeCK Analysis of CRISPR Screens. Related to Fig 2. This S1 Table presents the sgRNA sequences utilized for the construction of the ovine genome-wide CRISPR library.
(XLSX)

S2 Table MAGeCK Analysis of CRISPR Screens. Related to Fig 3. This S2 Table presents the sequencing results for sgRNAs targeting sequences within CRISPR-knockout, sorted mutant cell populations that contain the complete sgRNA library.
(XLSX)

S3 Table Gene ontology analysis of 0.1% of the ranked hits from the result of the MAGeCK analysis. Related to Fig 3. This S3 Table presents the Gene Ontology (GO) analysis of 0.1% of the ranked hits from the MAGeCK analysis results, highlighting enriched biological processes, molecular functions, or cellular components associated with the identified host genes in the context of ORFV screening.
(XLSX)

S4 Table Primers used in this research and related to STAR Methods. This S4 Table lists the primers used in this research, corresponding to the details described in the Methods.
(XLSX)
