# Supplementary figures and images for: Genome-wide CRISPR screen reveals PEX11B as a host restriction factor against ORFV through membrane fluidity regulation
Source: PLoS Pathog. 2026 Jul 15;22(7):e1013767. doi: 10.1371/journal.ppat.1013767 (PMC13387614; doi:10.1371/journal.ppat.1013767)

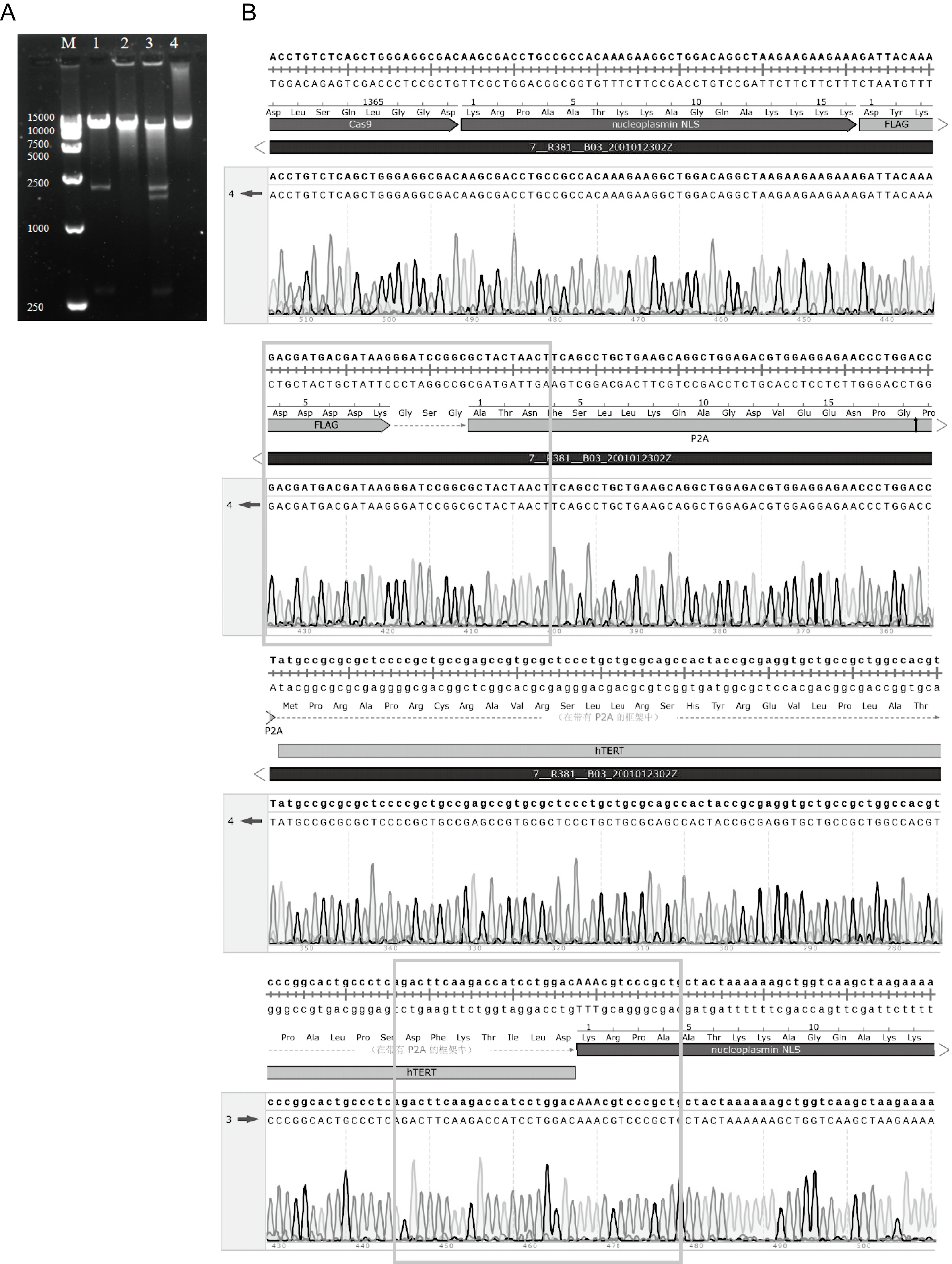

Supplement: S1 Fig — (A) Restriction enzyme digestion identification of recombinant pLV-Cas9-hTERT plasmid. M: DL15,000 DNA molecular weight marker; Lane 1: pLV-Cas9-hTERT digested with BsmBI (329 bp, 1994 bp and 14064 bp fragments); Lane 2: pLV-Cas9-hTERT linearized by single EcoRI digestion (single 16387 bp band); Lane 3: pLV-Cas9-hTERT double-digested with BsmBI and EcoRI (329 bp, 1994 bp, 1645 bp and 12419 bp fragments); Lane 4: undigested intact pLV-Cas9-hTERT plasmid. (B) Sanger sequencing verification of recombinant pLV-Cas9-hTERT plasmid. Representative sequencing chromatograms of the inserted fusion fragment from pLV-Cas9-hTERT construct. Regions highlighted by boxes correspond to the sequencing reads spanning the recombinant ligation junctions of the target insertion. (TIF) [file ppat.1013767.s001.tif]

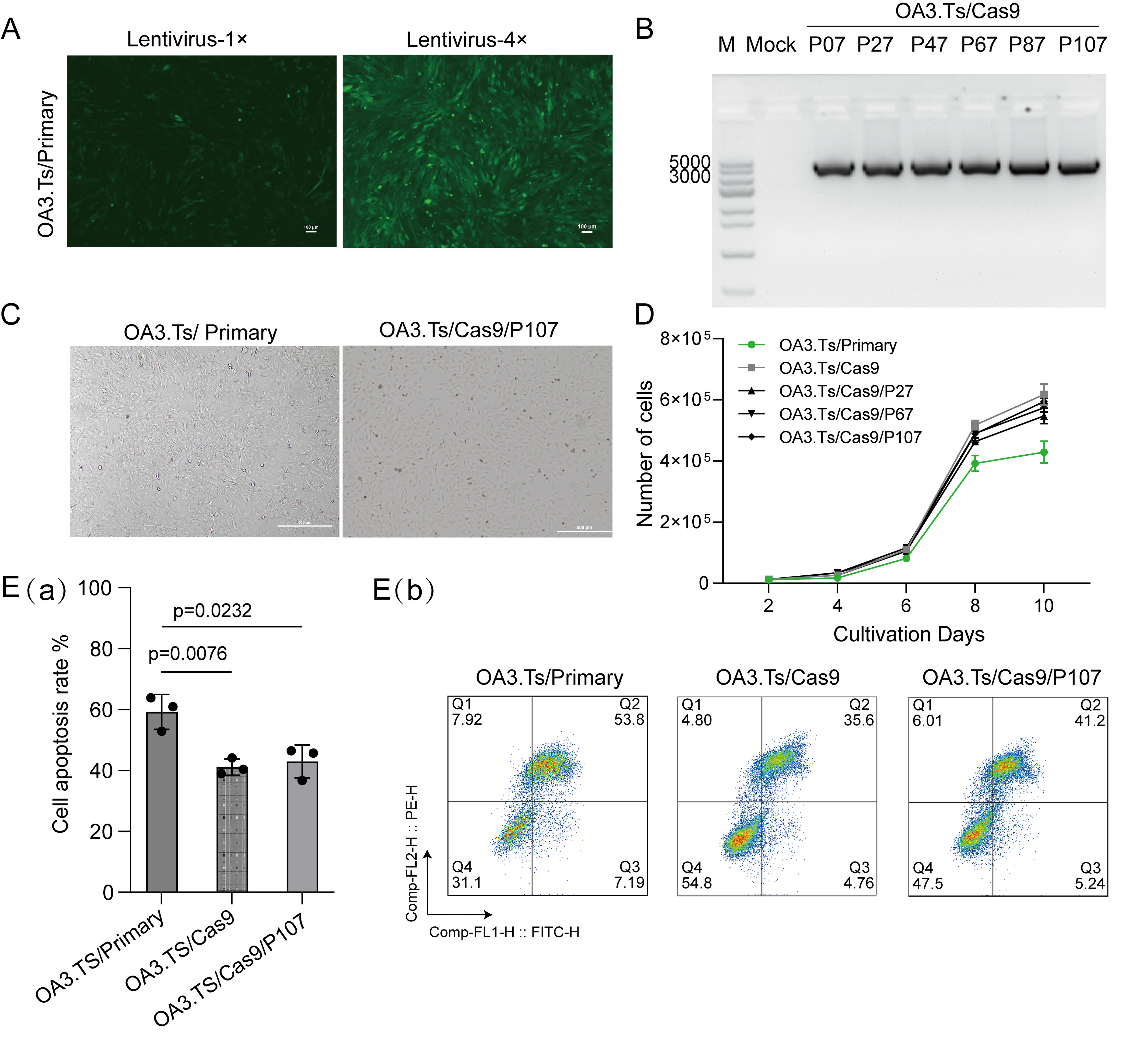

Supplement: S2 Fig — (A) eGFP expression in OA3.Ts primary cells transduced with different concentrations of lentivirus. Primary OA3.Ts cells were infected with eGFP-encoding lentivirus at either 1× (undiluted lentiviral stock) or 4×(four-fold concentrated lentiviral stock). Scale bar = 100 μm. (B)PCR detection of Cas9-hTERT fragment in OA3.Ts/Cas9 cells at different passage numbers. Genomic DNA was extracted from OA3.Ts/Cas9 cells at indicated passage numbers (P07, P27, P47, P67, P87, P107). Mock, untreated primary cells. (C) Morphological comparison between primary OA3.Ts cells and OA3.Ts/Cas9 cells at late passage. Representative phase-contrast (or brightfield) images showing the morphology of primary OA3.Ts cells and OA3.Ts/Cas9 cells at passage 107 (P107). Scale bar = 500 μm. (D) Proliferation curves of OA3.Ts primary cells and OA3.Ts/Cas9 cells at different passage numbers. (E) Detection of apoptosis efficiency in cells of different passage generations(a)Summary chart of apoptosis efficiency under different passage numbers.(b)Flow cytometry plots of apoptosis efficiency at different cell passage numbers.Vertical axis (PE-H): Annexin V-PE fluorescence, indicating phosphatidylserine externalization (early apoptosis).Horizontal axis (Comp-FL2-H): PI fluorescence, reflecting loss of membrane integrity (late apoptosis/necrosis).Quadrant analysis:Q1 (upper left): Necrotic or late apoptotic cells (Annexin V − / PI⁺).Q2 (upper right): Late apoptotic cells (Annexin V ⁺ / PI⁺).Q3 (lower right): Early apoptotic cells (Annexin V ⁺ / PI−).Q4 (lower left): Viable cells (Annexin V − / PI−). (TIF) [file ppat.1013767.s002.tif]

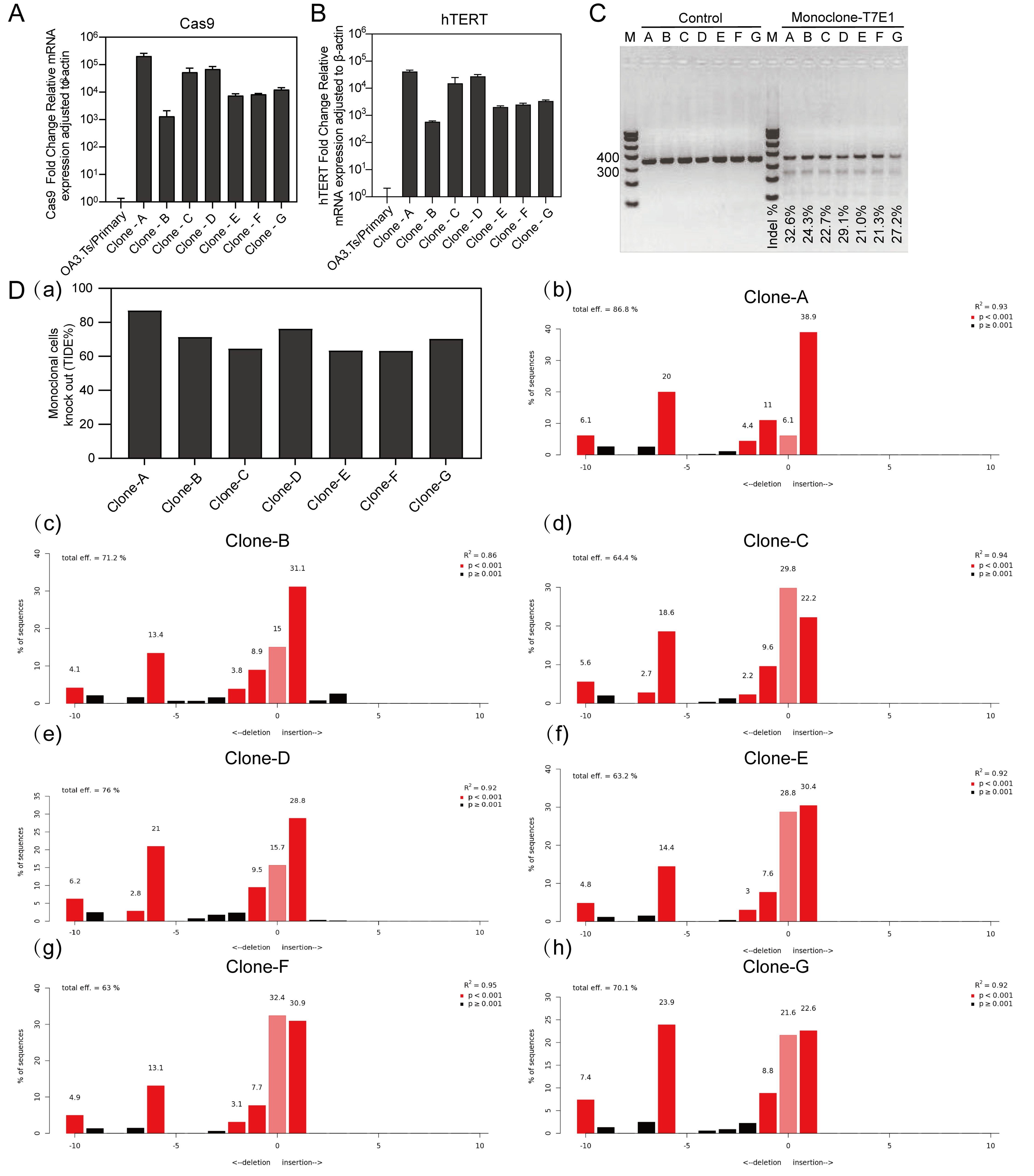

Supplement: S3 Fig — (A) Cas9 mRNA expression levels in OA3.Ts/Cas9 clonal cell lines measured by qPCR. Total RNA was extracted from seven OA3.Ts/Cas9 clonal cell lines (Clone A through Clone G), and reverse-transcribed to cDNA. Cas9 gene expression was quantified by quantitative real-time PCR (qPCR) and normalized to the housekeeping gene β-actin. Each bar represents mean ± SEM (or SD) from at least three technical replicates. (B) hTERT mRNA expression levels in OA3.Ts/Cas9 clonal cell lines measured by qPCR. (C) Evaluation of Cas9 activity among candidate single-cell-derived clones using a T7EN I cleavage assay. The candidate cells were transduced with a validated sgRNA (targeting the B4GALNT2 gene) lentivirus. The single-cell-derived clone with the highest Cas9 activity is Clone#A. Indels% indicates the percentage of alleles with insertions or deletions (indels) at the target locus, determined by T7EI mismatch cleavage assay. (D) TIDE analysis of indel efficiency in monoclonal cells derived from CRISPR/Cas9-edited OA3.Ts cells. (a) Bar graph showing the percentage of monoclonal cells with knockout efficiency in seven isolated clones (Clone-A to Clone-G) as determined by TIDE analysis. (b-h) Indel spectra of individual clones analyzed by TIDE. Red bars represent insertion/deletion (indel) frequencies; the total editing efficiency and goodness-of-fit (R2) for each clone are indicated. Statistical significance of indel events is denoted by color (p < 0.001 vs. control). (TIF) [file ppat.1013767.s003.tif]

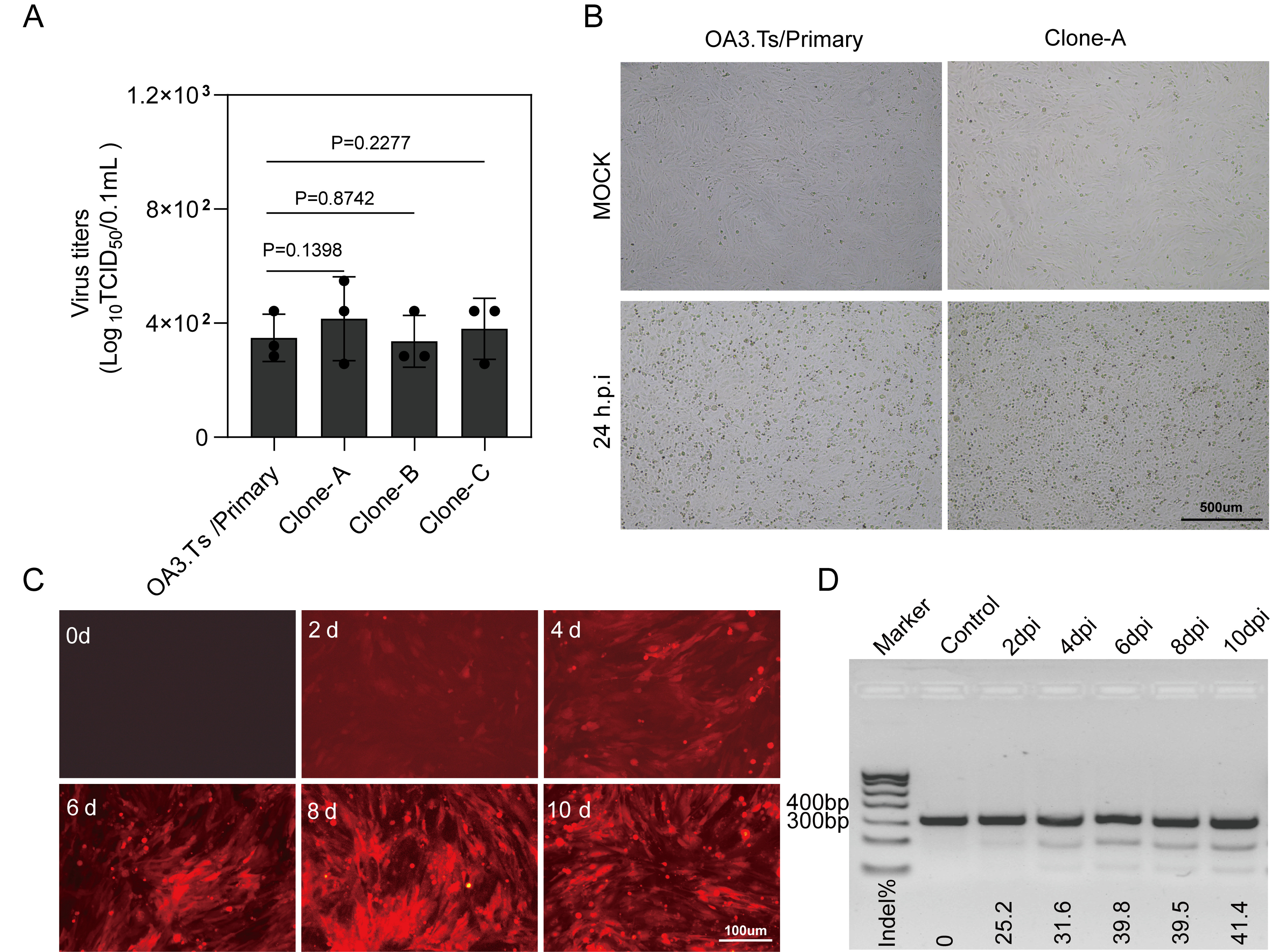

Supplement: S4 Fig — (A) Cas9 and hTERT expression does not affect ORFV infection in OA3.Ts/cas9 clone. ORFV virus titers in parental OA3.Ts primary cells and three OA3.Ts/Cas9 monoclonal clones (Clone-A, Clone-B, Clone-C) were determined by TCID50 assay at 24h post-infection with an MOI of 0.001. (B) Assessment of the infection efficiency of sgRNA lentivirus in OA3.Ts/cas9/clone#A cells at 24h post-infection with an MOI of 0.001. (C) Assessment of gene editing efficiency in OA3.Ts/Cas9/clone#A cells using the T7E1 endonuclease digestion assay. Results indicate that the gene editing efficiency tends to stabilize at 6 days following lentiviral infection. Scale bar,100um. (D) Assessment of the cleavage activity of sgRNA lentivirus in OA3.Ts/cas9/clone#A cells at the time points indicated using a T7EN I assay. Indel%: percentage of indels; bp: base pairs; dpi: days post infection, Control: wild-type cells; Marker: Marker I DNA ladder. (TIF) [file ppat.1013767.s004.tif]

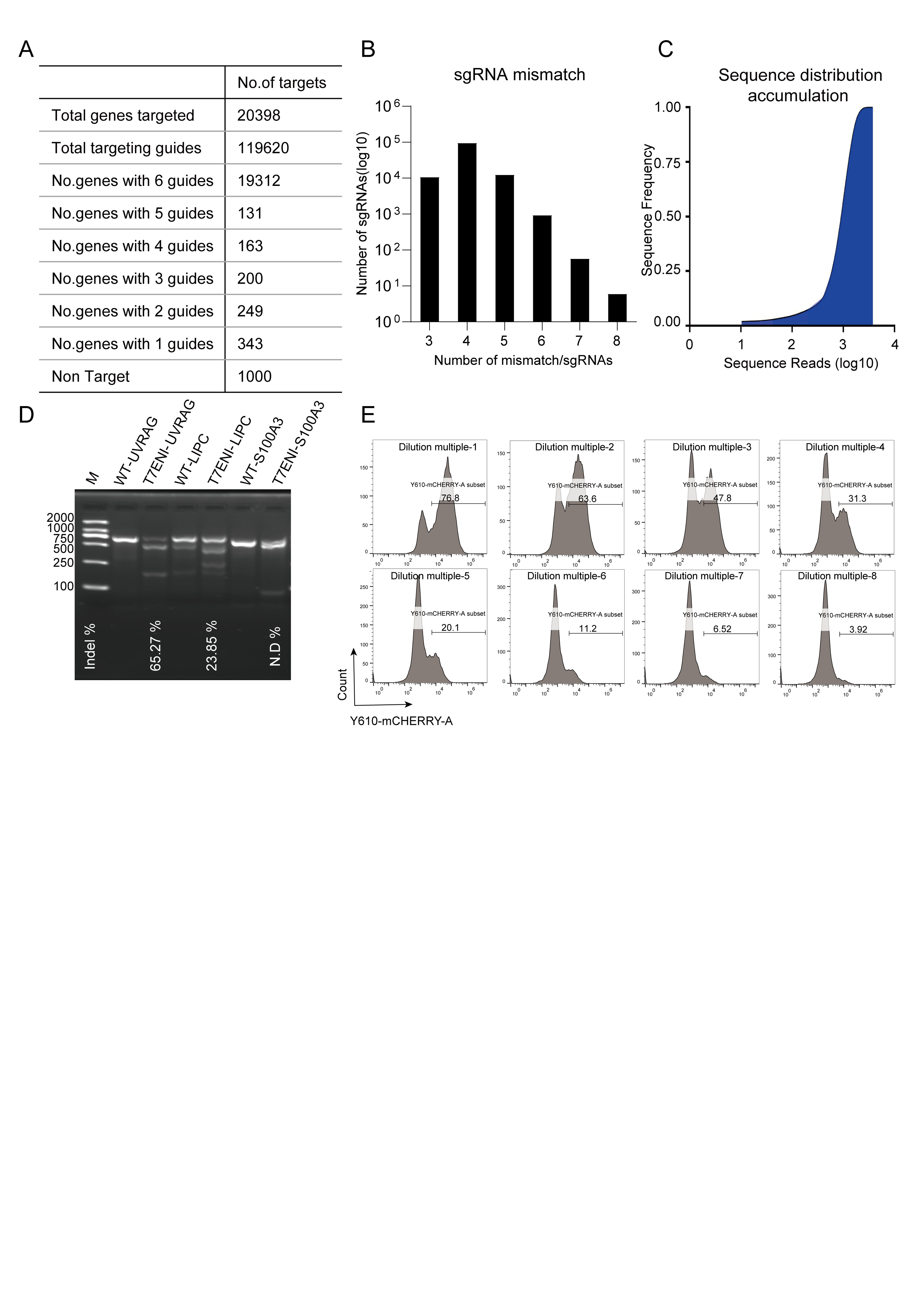

Supplement: S5 Fig — (A) Overview table of distribution of ovine genome-wide sgRNA Library. The library comprises a total of 119,620 targeting guide RNAs (sgRNAs) directed against 20,398 protein-coding genes. Among these, 19,312 genes are targeted by 6 guides per gene, while the remaining genes are covered by 5–1 guide(s) (131, 163, 200, 249, and 343 genes for 5, 4, 3, 2, and 1 guide(s), respectively). Additionally, 1,000 non-targeting control guides are included. The design ensures broad and balanced coverage for genome-scale functional screening applications. (B) The count of mismatches for each sgRNA in the library. The information is derived from the CRISPR sgRNA library of the sheep whole genome, which is utilized to evaluate the targeting precision and off-target potential of sgRNAs. (C) Sequencing read distribution accumulation of the sgRNA plasmid library. The majority of sgRNAs exhibit read counts between 1.5 and 3.5 log10, with a peak around 3.0-3.5, indicating balanced library representation. (D) Evaluation of knockout effects of a randomly selected sgRNA (UVRAG,LIPC,S100A3) from the initially designed sgRNA library by T7EI assay. (E) Analysis of the positive rate of cells following lentiviral infection (with 5-fold serial dilution) using flow cytometry. (TIF) [file ppat.1013767.s005.tif]

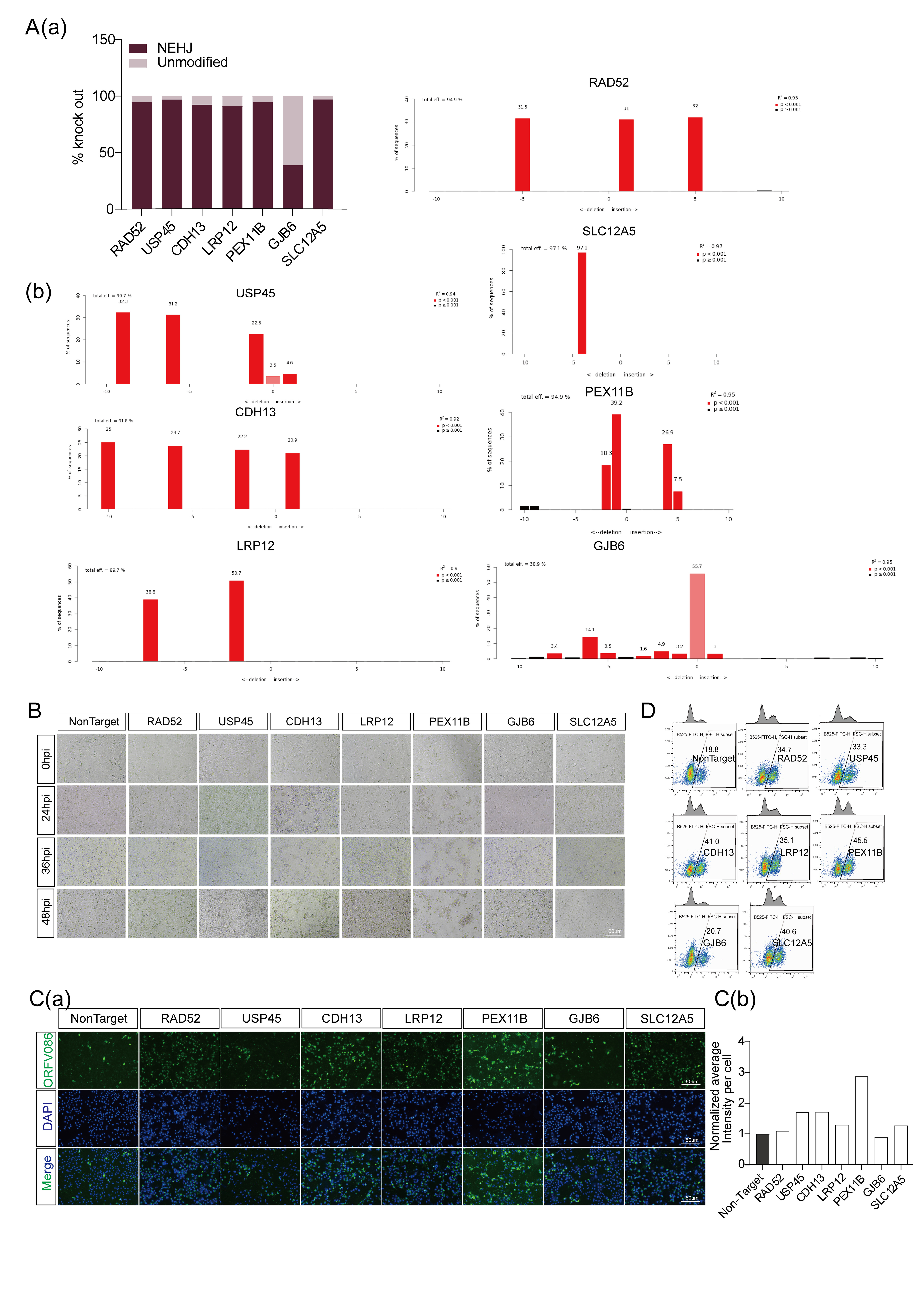

Supplement: S6 Fig — (A) CRISPR knockout efficiency analysis of candidate genes.(a) Bar graph showing the percentage of gene knockout in NEHJ and unmodified groups for the top candidate genes identified in the CRISPR screen.(b) Indel spectra of each candidate gene analyzed by TIDE. Red bars represent the frequency of insertion/deletion (indel) events, with total editing efficiency and goodness-of-fit (R2) indicated for each target. Statistical significance of indel frequencies is denoted by color (p < 0.001 vs. control). (B) The CPE (cytopathic effect) of seven-knockout cell lines infected at different time points. (MOI = 2) Scale bar = 100 μm. (C) (a) Representative immunofluorescence images of seven-knockout cell lines infected with ORFV (MOI = 2) for 48 h. Scale bar = 50 μm. (b)The normalized average intensity of ORFV V086 signal per cell, with the Non target control set to 1. Data are representative of three independent biological replicates. (D) Flow cytometric analysis of ORFV086 expression in seven ORFV-infected knockout polyclonal cell and Non target cell lines related to Fig 3H. (TIF) [file ppat.1013767.s006.tif]

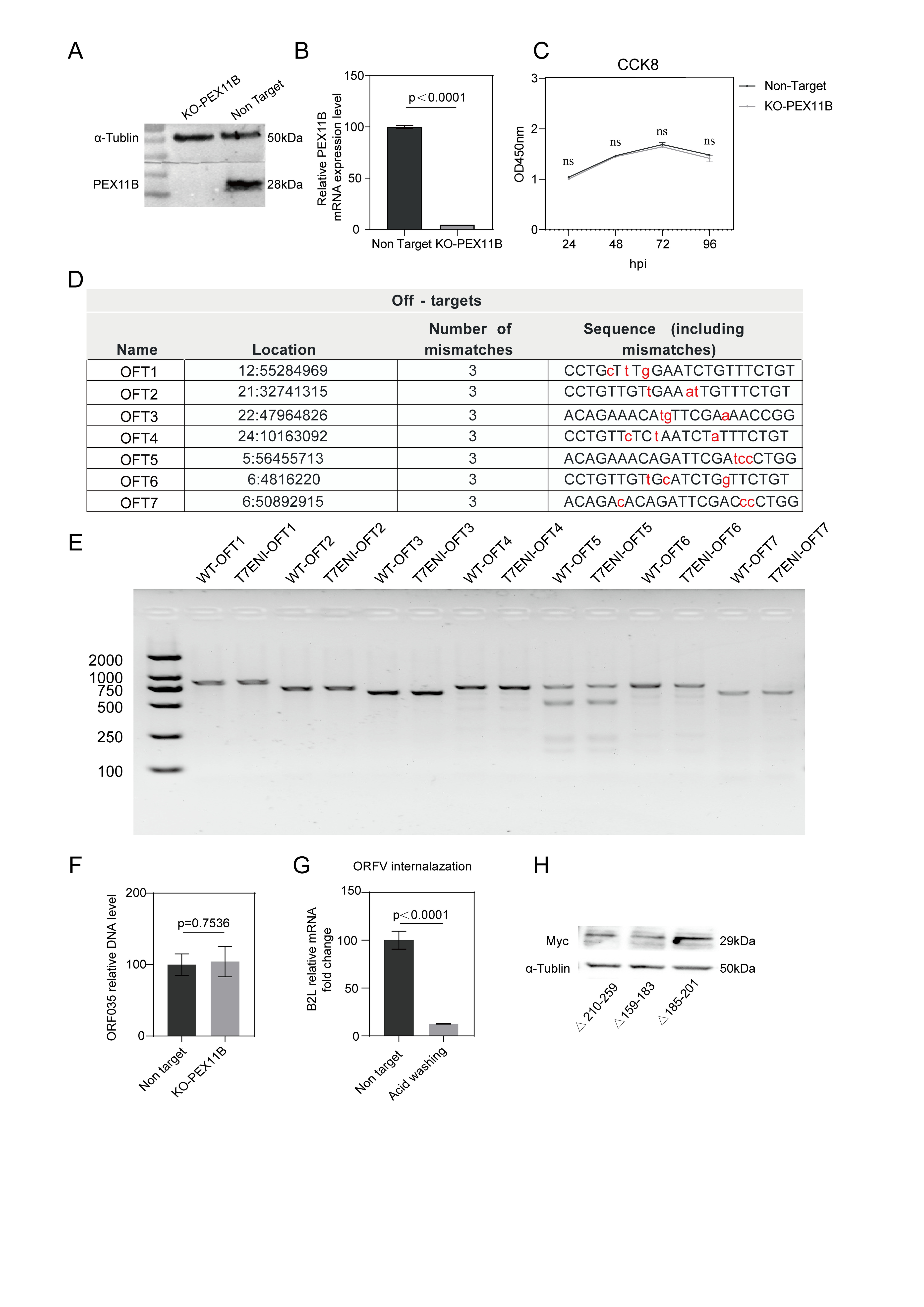

Supplement: S7 Fig — (A) Western blot assay to detect the PEX11B protein expressed in KO-PEX11B and Non Target cells. α-Tublin used as an internal control gene. (B) Relative quantitative real-time PCR detection of PEX11B expression levels in KO-PEX11B and Non Target cells. (C) Cell viability in KO-PEX11B versus Non target cells by cell counting kit-8 assay. (D) Table listing the top seven predicted off-target sites (OFT1-OFT7) with up to 3 mismatches. Genomic location, number of mismatches, and sequences (with mismatches highlighted in red) are shown. (E) T7 endonuclease I (T7EI) assay of the seven predicted off-target sites. No cleavage products indicative of off-target editing were detected in the KO-PEX11B cell line, confirming the absence of detectable off-target mutations. WT, wild-type. (F) Quantitative analysis of ORFV binding was performed by relative quantification targeting the early ORFV gene ORF035. (G) Elution efficiency was detected using pH = 3.0 PBS. Data are presented as means ± SD (n = 3). (H) Western blot analysis of Myc-tagged PEX11B truncation mutants.Expression of Myc-tagged PEX11B truncation mutants (Δ210–259, Δ159–183, and Δ185–201) was detected by immunoblotting using an anti-Myc antibody. α-Tublin was used as a loading control. The Myc-tagged proteins migrated at the expected size of ~29 kDa, and α-Tublin at ~50 kDa, confirming successful expression of each truncation construct. (TIF) [file ppat.1013767.s007.tif]

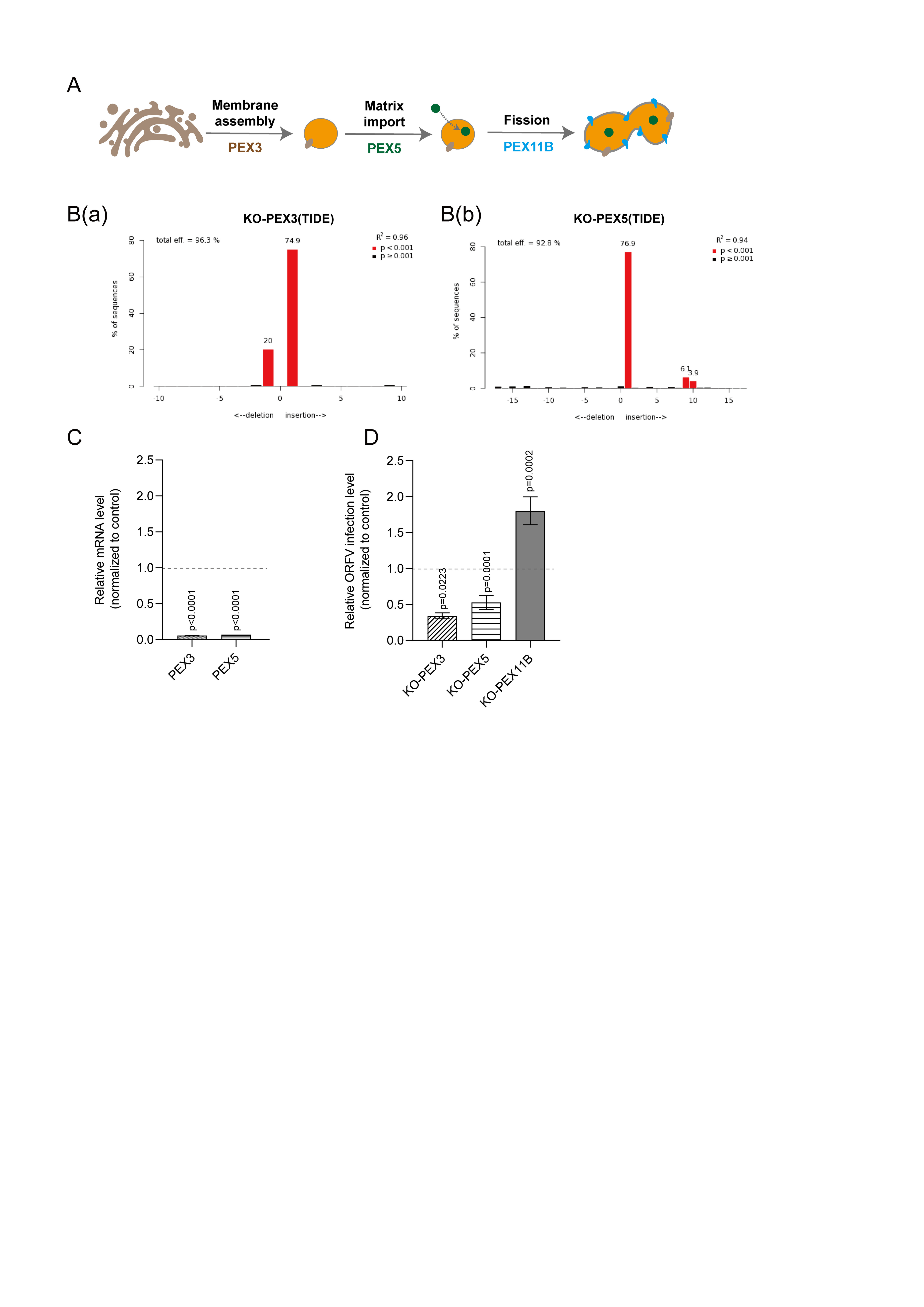

Supplement: S8 Fig — (A) Schematic illustration of peroxisome formation: PEX3 mediates membrane assembly, PEX5 mediates matrix protein import, and PEX11B controls organelle fission. (B) TIDE analysis of CRISPR-edited cells to validate PEX3 (a) and PEX5 (b) knockout efficiency. X-axis represents indel size (deletions < 0, insertions > 0); Y-axis shows the proportion of sequencing reads for each indel. Total editing efficiency and goodness-of-fit (R2) are displayed for each sample; significant indels (p < 0.001) are colored red. (C) Quantitative RT-PCR analysis validates efficient knockdown of PEX3 and PEX5 in respective knockout cell lines. Relative mRNA abundance of PEX3 in PEX3-knockout cells and PEX5 in PEX5-knockout cells was normalized to nontarget control cells (dashed line set to 1). (D) Relative ORFV infection levels in KO-PEX3, KO-PEX5, and KO-PEX11B cells normalized to Non Target cells. p-values reflect statistical comparisons versus control. (TIF) [file ppat.1013767.s008.tif]

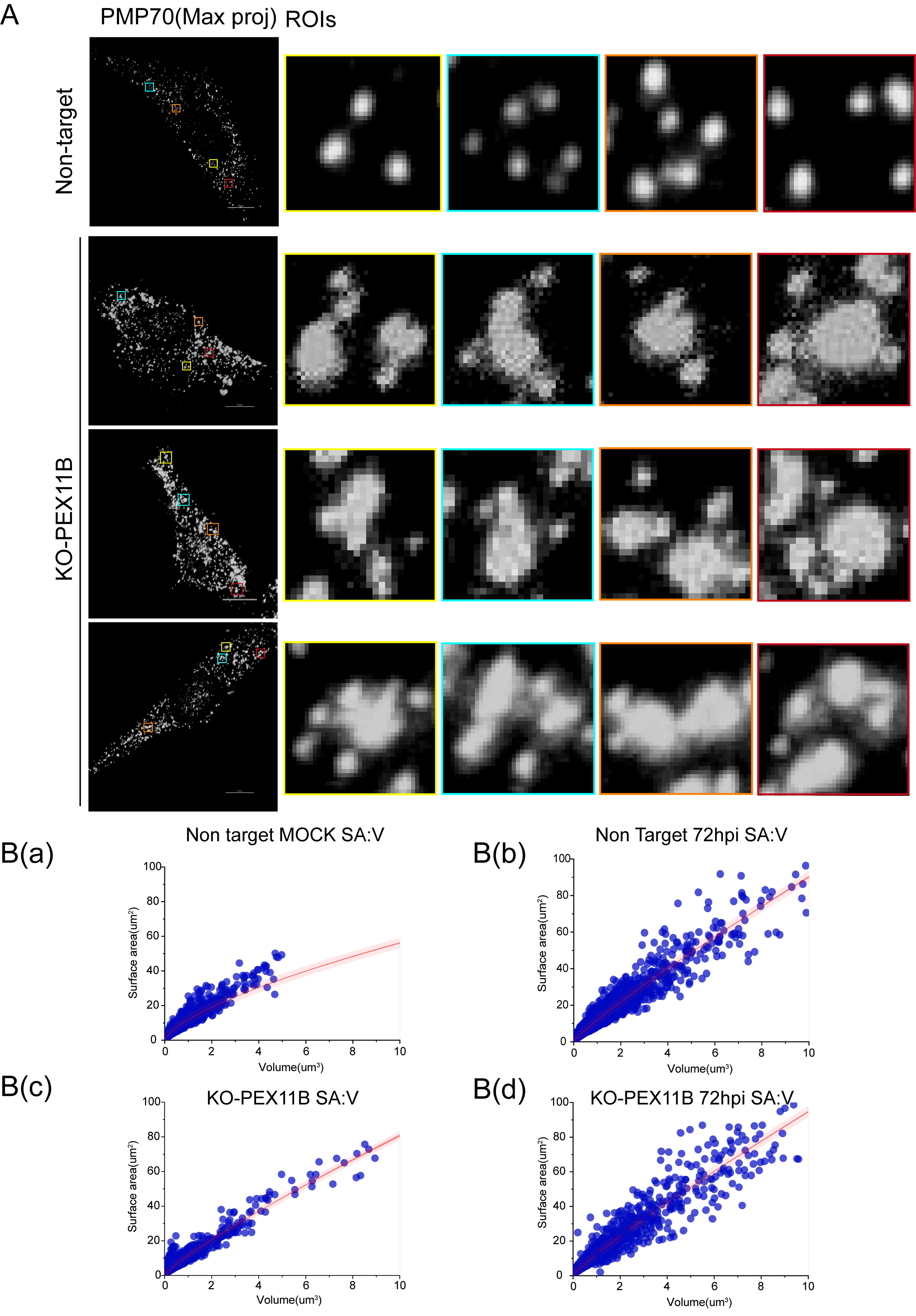

Supplement: S9 Fig — (A) Maximum projections of Non target and KO-PEX11B cells with anti-PMP70 and imaged at 100X. A square frame indicates ROIs in the corresponding color. Scale bars = 10 µm. (B) Plot of the surface area and volume of individual peroxisomes in (a) Non Target Mock cells (mean SA/V = 24.83 μm-1), (b) Non target 72hpi cells (mean SA/V = 31.83 μm-1),(c) KO-PEX11B (mean SA/V = 27.34 μm-1),(d) KO-PEX11B 72hpi cells (mean SA/V = 32.86 μm-1). Solid line indicates the regression curve, dashed lines indicate the upper and lower 95% confidence intervals. (TIF) [file ppat.1013767.s009.tif]

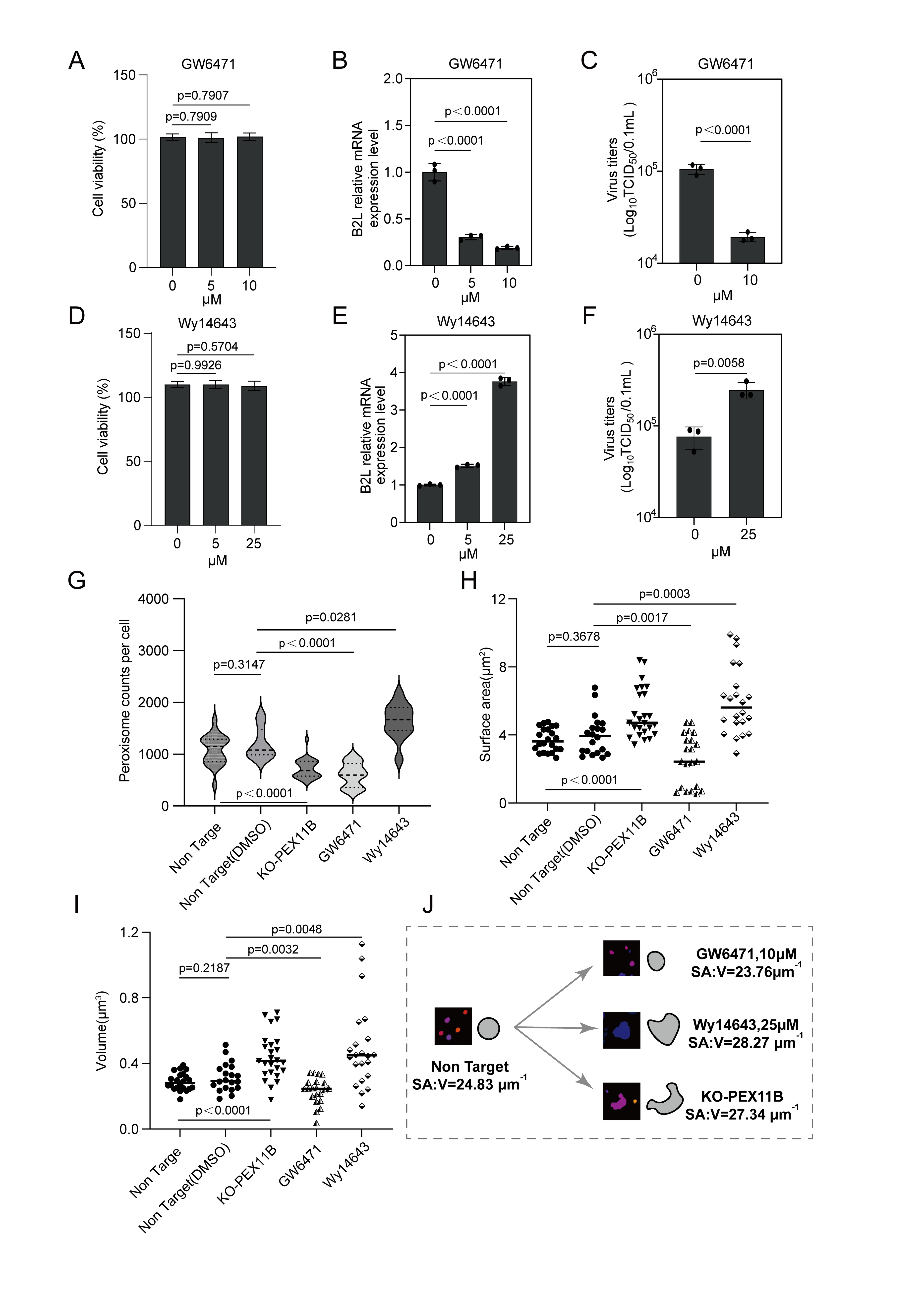

Supplement: S10 Fig — (A) Cell viability of Non target cells treated with GW6471 was assessed using the Cell Counting Kit-8. (B,C) ORFV infectious B2L gene mRNA expression level and virus titer produced from infected OA3.Ts/Cas9 cells following GW6471 treatment to inhibit peroxisome biogenesis. (D) Cell viability of Non target cells treated with Wy14643 was assessed using the Cell Counting Kit-8. (E,F) ORFV infectious B2L gene mRNA expression level and virus titer produced from infected OA3.Ts/Cas9 cells following Wy14643 treatment to induce peroxisome biogenesis. (G) Quantification of peroxisome number per cell under different group. Peroxisomes were counted via immunofluorescence staining of peroxisomal membrane marker PMP70 in Non target cells,Non target cells(vehicle DMSO), KO-PEX11B cells, Non target cells with GW6471 10 μM and Non target cells with Wy14643 25 μM. (H,I) The average volume of peroxisomes per cell is shown in (H), the average surface area of peroxisomes per cell is shown in (I). N = 22 cells in Non Target Mock, N = 22 cells in Non Target DMSO,N = 27 in KO-PEX11B Mock, N = 22 in Non Target with GW6471 10 μM, N = 24 in Non Target with Wy14643 25 μM. (J) Schematic representation of peroxisome morphological changes induced by different manipulations. Peroxisome surface area-to-volume (SA/V) ratios and representative shapes are shown for Non target cells, GW6471-treated cells (10 μM), Wy14643-treated cells (25 μM), and KO-PEX11B cells. (TIF) [file ppat.1013767.s010.tif]

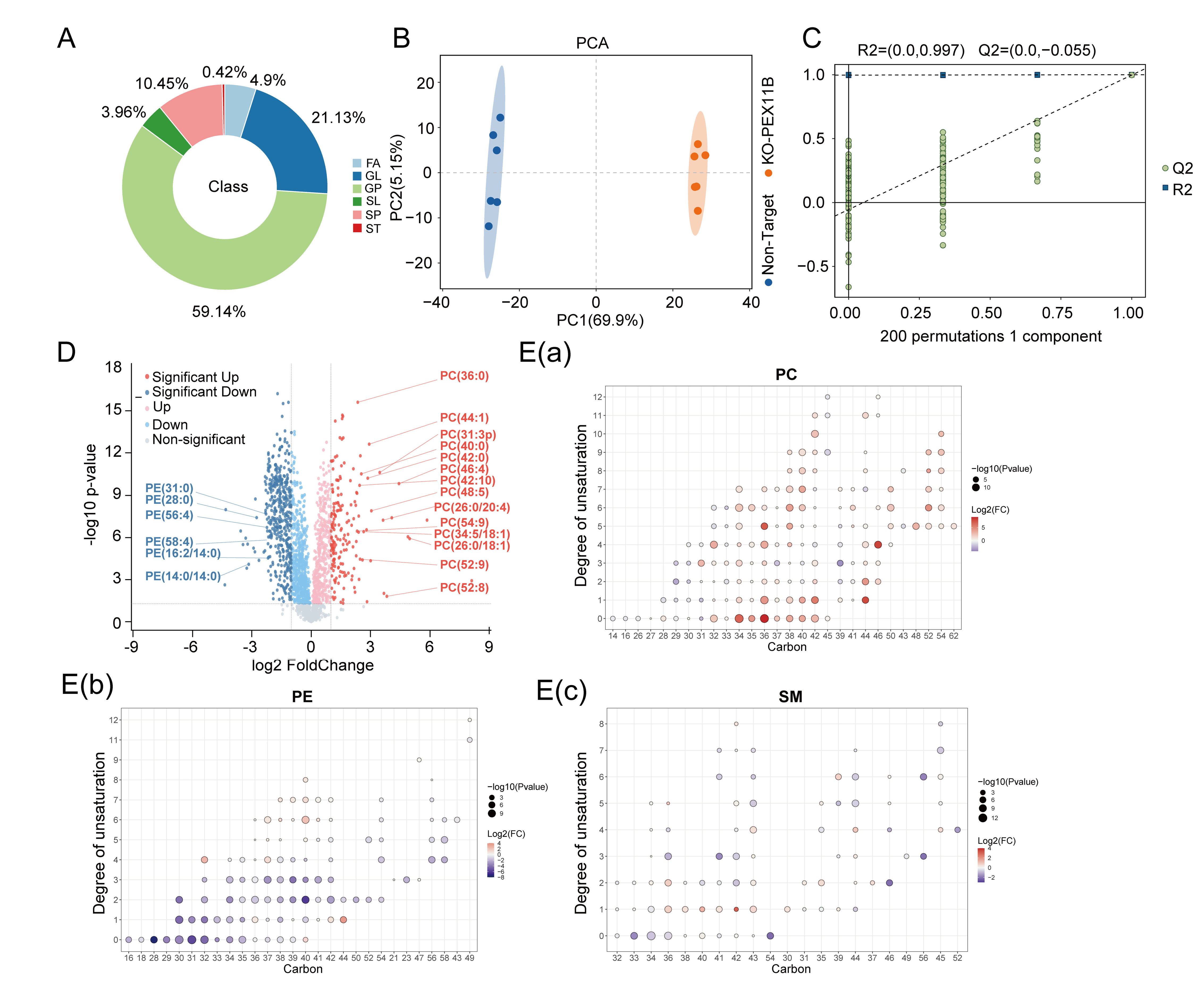

Supplement: S11 Fig — (A) Statistical summary of lipidomics categorization of lipids in cells. Lipid relative abundances were quantified based on peak area normalization. Each segment represents the percentage contribution of an individual lipid class: FA (fatty acids, 4.9%), GL (glycerolipids, 21.13%), GP (glycerophospholipids, 59.14%), SL (sphingolipids, 3.96%), SP (sterol lipids, 10.45%), and ST (steroids, 0.42%). (B) Principal component analysis (PCA) of lipid profiles distinguishes Non Target and KO-PEX11B cells. PCA score plot based on global lipidomic data showing clear separation between the Non Target group (blue dots) and KO-PEX11B group (orange dots). Ellipses denote the 95% confidence interval for each group. (C) Permutation test (n = 200) validating the OPLS-DA model. The plot displays R2 (green triangle) and Q2 (blue square) values obtained after 200 random permutations of the Y matrix. The original model’s R2 = 0.997 and Q2 = -0.055 (red symbols) fall outside the permutation distribution, indicating that the observed separation is not due to over-fitting and the model possesses robust predictive power. (D)Differential lipid distribution volcano plot. Red dots represent metabolites that are upregulated in the experimental group, blue dots represent downregulated metabolites, and gray dots represent metabolites that are not significant. The horizontal axis shows the log2(FC) values for the comparison between the two groups, while the vertical axis represents -log10(p-value) values. Individual significant differences in lipids have been annotated. (E) A classified bubble chart showing the relationship between carbon chain length and unsaturation in PC(a), PE (b), and SM(c). The x-axis represents the carbon chain length of the lipids, while the y-axis represents the unsaturation of the lipids. The color of the bubbles maps to Log2(FC), and larger circles indicate smaller p-value values. (TIF) [file ppat.1013767.s011.tif]

Fig .1B

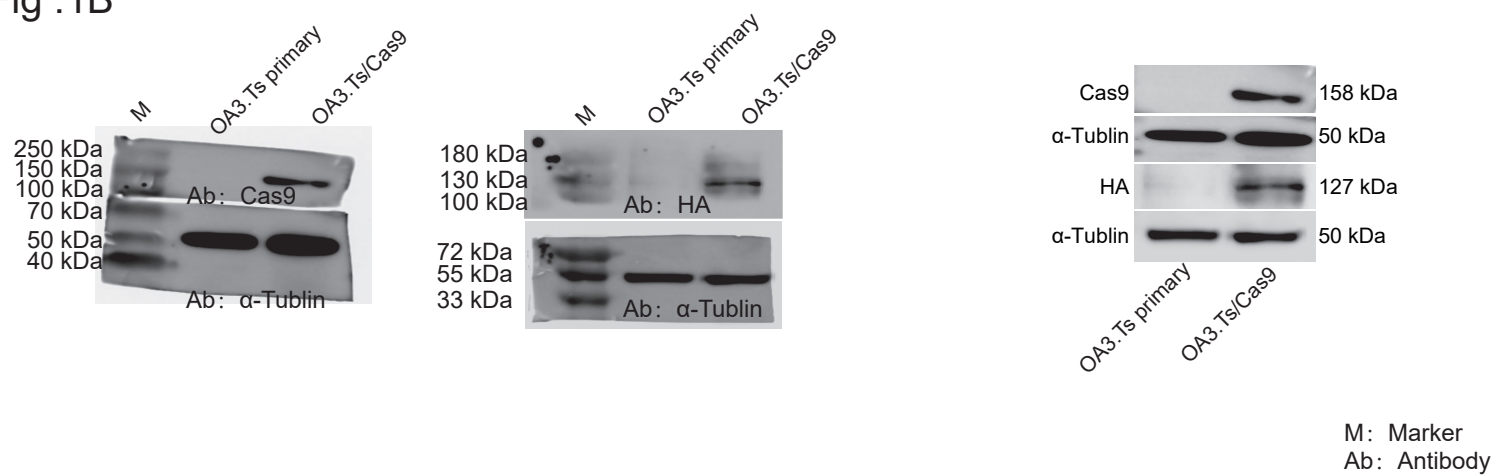

Fig .4J

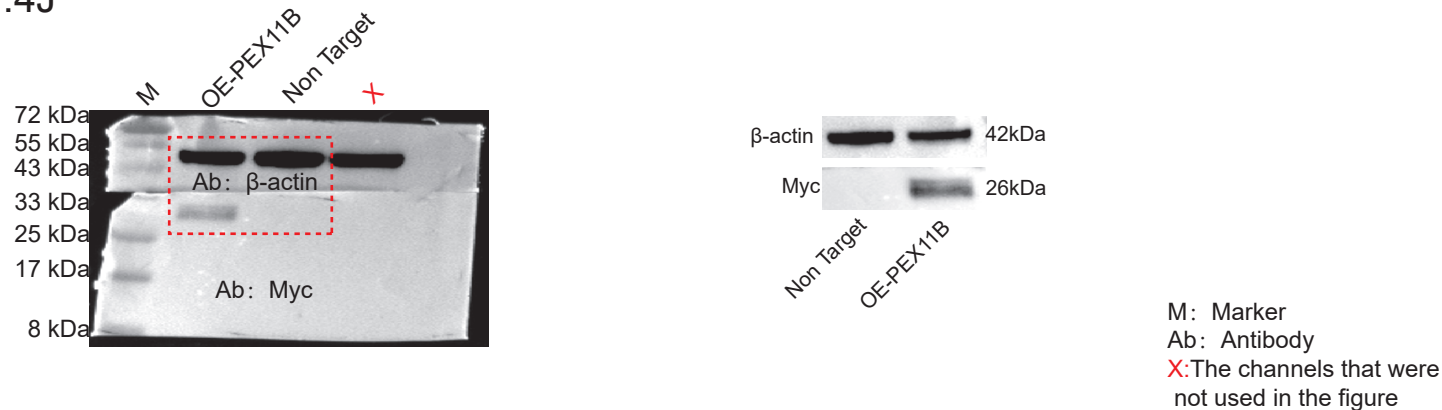

Fig .S7A

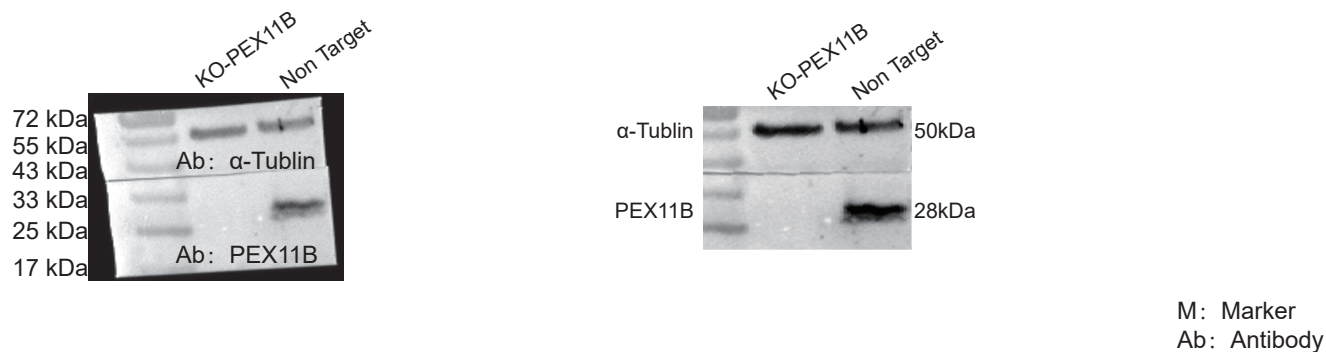

Fig .S7H

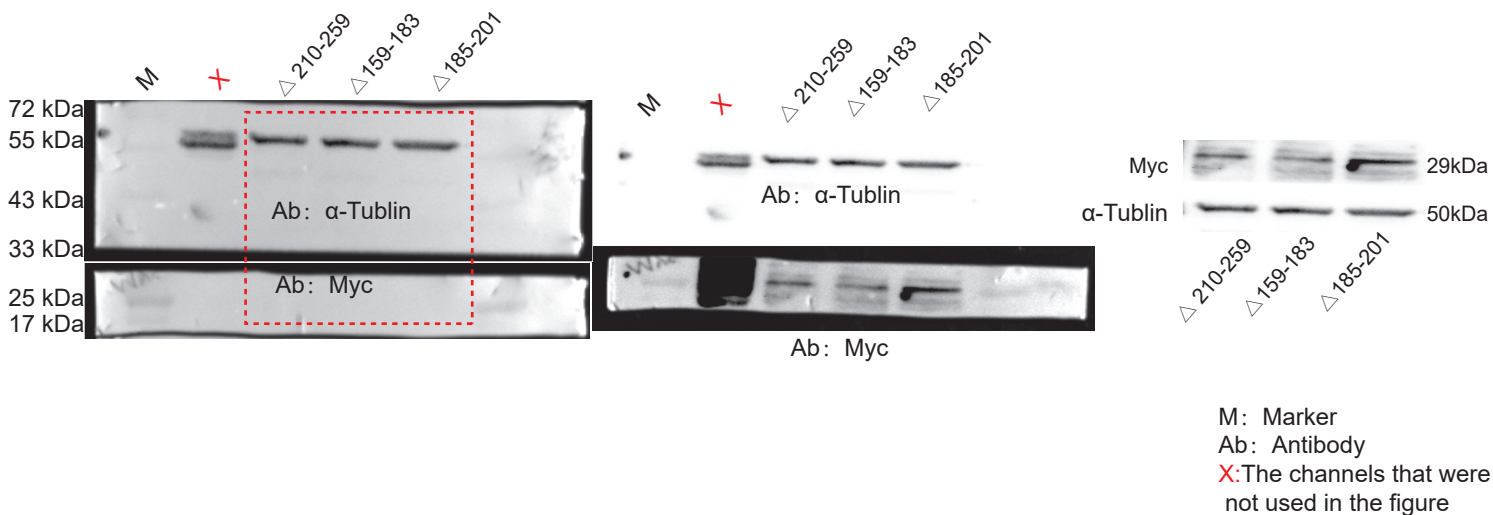

Supplement: S1 File — Uncropped original western blot images corresponding to Fig 1B, Fig 4J, Fig S7A and Fig S7H. (PDF) [file ppat.1013767.s012.pdf]
